# Deoxyguanosine kinase deficiency couples purine metabolism to innate immune activation and lipid accumulation in hepatocytes

**DOI:** 10.3389/fimmu.2026.1758569

**Published:** 2026-07-15

**Authors:** Maija Corey, Mahati Rayadurgam, Mousa Vatanmakanian, Alicia Gibbons, Carolina Altbaum, Jeamin Jung, Neha Reddy, Priyanka Saminathan, Kiyokazu Kakugawa, Sonia Sharma

**Affiliations:** 1 Center for Autoimmunity and Inflammation, La Jolla Institute for Immunology, La Jolla, CA, United States; 2 Department of Medicine, University of California, San Diego, San Diego, CA, United States; 3 Laboratory for Inflammatory Immune Metabolism, Center for Integrative Medical Sciences, RIKEN, Yokohama, Japan

**Keywords:** deoxyguanosine kinase deficiency, hepatic steatosis, human endogenous retroviral elements, immunometabolism, purine metabolism, type I interferon

## Abstract

**Introduction:**

Mitochondrial DNA depletion syndromes (MDS) caused by deoxyguanosine kinase (DGUOK) deficiency are classically attributed to impaired mitochondrial DNA (mtDNA) maintenance. However, many patients develop hepatic steatosis and inflammation despite preserved mtDNA content, suggesting that additional pathogenic mechanisms contribute to disease. DGUOK is a key enzyme in the mitochondrial purine salvage pathway, but its role in coordinating purine metabolism with lipid homeostasis and innate immune signaling remains poorly understood.

**Methods:**

Acute DGUOK deficiency was induced in human hepatocellular carcinoma (HepG2) hepatocytes by siRNA-mediated knockdown. Mitochondrial integrity was assessed by mtDNA quantification, mitochondrial morphology, and oxidative phosphorylation (OXPHOS) protein expression. Lipid accumulation was evaluated by BODIPY staining, and transcriptomic changes were analyzed by bulk RNA sequencing. Purine imbalance was modeled by treatment of wild-type cells with 2′-deoxyadenosine, followed by assessment of DNA methylation, interferon signaling, and lipid accumulation.

**Results:**

Acute DGUOK depletion induced a 2.9-fold increase in intracellular lipid droplet accumulation and activation of a type I interferon (IFN) transcriptional program despite preserved mtDNA copy number, mitochondrial morphology, and OXPHOS complex expression. Bulk RNA sequencing revealed induction of human endogenous retroviruses (HERVs) and interferon-stimulated genes (ISGs), together with suppression of lipid metabolic pathways and remodeling of purine-, methionine-, and methylation-associated networks. Consistent with these transcriptional changes, DGUOK-deficient cells exhibited an approximately 40% reduction in global DNA methylation, accompanied by hypomethylation of CpG-rich region within the *ISG15* and *ISG20* promoters. Perturbation of purine homeostasis with exogenous 2′-deoxyadenosine phenocopied DGUOK deficiency, driving DNA hypomethylation, activation of viral mimicry pathways, and lipid accumulation.a

**Discussion:**

These findings demonstrate that acute DGUOK deficiency promotes innate immune activation and metabolic reprogramming through a purine-dependent mechanism that precedes mtDNA depletion and overt mitochondrial dysfunction. By linking disrupted mitochondrial purine salvage to HERV and ISG derepression, interferon signaling, epigenetic remodeling, and steatosis, this study provides a mechanistic framework for the immunometabolic pathology of DGUOK deficiency and identifies mitochondrial purine metabolism as an important regulator of hepatic immune and metabolic homeostasis.

## 1 Introduction

Mitochondrial DNA (mtDNA) depletion syndromes (MDS) comprise a genetically and clinically heterogeneous group of autosomal-recessive disorders characterized by impaired mitochondrial nucleotide homeostasis, reduced mtDNA copy number, and progressive dysfunction in high-energy tissues such as the liver, muscle, and central nervous system (1, 2). As mtDNA encodes essential subunits of the oxidative phosphorylation (OXPHOS) machinery, defects in mitochondrial nucleotide metabolism compromise respiratory capacity and lead to hepatic failure, neuromuscular abnormalities, and multisystem metabolic stress (1, 3). Histopathological analyses of liver biopsies from infants with MDS frequently reveal condensed mitochondrial cristae and striking lipid droplet accumulation, underscoring the close integration of mitochondrial nucleotide balance, energy production, and hepatocellular metabolic homeostasis (4, 5).

Deoxyguanosine kinase (DGUOK) is a central enzyme in the mitochondrial purine salvage pathway, catalyzing the phosphorylation of deoxyguanosine and deoxyadenosine to their monophosphate derivatives, which serve as precursors for mtDNA synthesis (6, 7). Loss-of-function mutations in DGUOK cause a severe hepatocerebral form of MDS that represents the majority of pediatric DGUOK deficiency cases (8, 9). Affected infants typically present with cholestasis, hepatomegaly, neurological impairment, and prominent hepatic steatosis that progresses to liver failure early in life (8, 10, 11). Although liver transplantation can restore hepatic function, systemic disease frequently persists or recurs, underscoring the multisystem nature of DGUOK-linked metabolic injury (11, 12). Given that no disease-modifying therapies exist for DGUOK deficiency, a deeper understanding of DGUOK’s cellular functions is essential for identifying actionable pathways and developing effective treatments.

Despite its canonical function in mtDNA replication, emerging clinical evidence suggests that mtDNA depletion alone does not fully explain the pathophysiology of DGUOK deficiency. Among more than 170 reported patients, fewer than half exhibit measurable mtDNA copy number reduction, yet many still develop steatosis and hepatic inflammation (6, 9, 10, 13, 14). Case reports describing genetically identical siblings with divergent clinical outcomes, including fulminant viral-induced liver failure in one twin but only mild steatosis in the other, further suggest that DGUOK dysfunction may sensitize hepatocytes to metabolic or immunologic stress independently of mtDNA loss (15). These findings support the existence of additional, as-yet unidentified metabolic or innate immune pathways disrupted in the early stages of DGUOK deficiency.

DGUOK functions within the mitochondrial arm of the purine salvage pathway (6, 7, 16, 17), placing it at a metabolic intersection that links nucleotide turnover, methyl-donor availability, and innate immune activation. Disruption of other purine-metabolizing enzymes, including adenosine deaminase (ADA1 and ADA2) or purine nucleoside phosphorylase (PNP), is known to induce spontaneous type I interferon responses through viral-mimicry mechanisms involving global DNA hypomethylation and derepression of human endogenous retrovirus (HERV) elements (18–20). These HERV-derived transcripts form double-stranded RNA-like structures sensed by RIG-I-like receptors or Toll-like receptor 3, triggering a cell-intrinsic innate immune type I interferon (IFN-I) response and interferon-stimulated gene (ISG) activation (18, 21–23). Purine imbalances can also alter methyl-donor metabolism, particularly *S*-adenosylmethionine (SAM) availability, which is essential for DNA and histone methylation as well as hepatic phosphatidylcholine synthesis and very-low-density lipoprotein (VLDL) secretion, processes intimately linked to lipid homeostasis (24–26).

These converging observations led us to hypothesize that DGUOK deficiency perturbs purine and methyl-donor metabolism in a manner that triggers innate immune activation and hepatocellular lipid accumulation independently of mtDNA loss. To test this, we used siRNA and Clustered Regularly Interspaced Short Palindromic Repeats/CRISPR-associated protein 9 (CRISPR/Cas9)-mediated silencing of DGUOK in HepG2 hepatocytes to investigate the immediate and long-term cellular consequences of DGUOK depletion, respectively. We assessed mtDNA content, mitochondrial structure and respiratory protein expression, quantified lipid accumulation, profiled transcriptome-wide changes in immune, metabolic and epigenetic pathways, and evaluated the effects of exogenous deoxyadenosine supplementation to model purine dysregulation. Our findings reveal an early, purine-driven immunometabolic program initiated by DGUOK loss, independent of mitochondrial genome depletion, which provides new mechanistic insight into the inflammatory and steatotic features of DGUOK deficiency. Delineating this immunometabolic program opens the door to therapeutic approaches that target purine balance, methyl-donor availability, or innate immune activation as potential actionable avenues to ameliorate disease in DGUOK deficiency.

## 2 Materials and methods

### 2.1 Cell culture

#### 2.1.1 Cell culture maintenance

HepG2 cells American Type Culture Collection (ATCC), Manassas, VA, USA were maintained in Eagle’s minimum essential medium (EMEM) (ATCC) with 10% heat-inactivated fetal bovine serum (FBS) and 100 U mL^−1^ penicillin–streptomycin, at 37°C, 5% CO_2_, and saturated humidity.

### 2.2 siRNA transfection

siGenome siRNA oligonucleotide pools were purchased from Dharmacon Lafayette, CO, USA, and transfections were performed using Lipofectamine™ RNAiMAX Transfection Reagent (Thermo Fisher Scientific Waltham, MA, USA) according to the manufacturer’s instructions with minor modifications optimized for HepG2 cells. Plates were precoated with poly-d-lysine (PDL) to enhance cell adherence and transfection efficiency.

For standard single-gene transfections, 6 pmol of siRNA were used per well of a 24-well plate and 120 pmol/100 mm dish. In the 24-well format, siRNA–lipid complexes were prepared in 100 µL Opti-MEM™ I Reduced Serum Medium (Thermo Fisher Scientific) with 1 µL Lipofectamine™ RNAiMAX and incubated for 10–20 min at room temperature before addition of 0.05 × 10^6^ cells in 400 µL antibiotic-free complete medium (final siRNA concentration = 10 nM). Reverse transfection was used for initial transfections, as it yielded higher efficiency in HepG2 cells. Cells were harvested at 48 h for RNA analysis, 72 h for protein and functional assays (including lipid quantification), and 96 h for mtDNA quantification, unless otherwise specified.

To rule out potential siRNA off-target effects, two individual DGUOK-targeting siRNAs (siDGk1 and siDGk2) were evaluated independently from the siDGUOK (siDGk) pool. HepG2 cells were transfected with siCONTROL (siCON), siDGk1, or siDGk2 using the same transfection conditions described above. Knockdown efficiency was assessed by RT-qPCR at 48 h and by Western blotting at 72 h posttransfection. To assess whether the independent siRNAs reproduce the same molecular and lipid phenotypes observed with the siDGk pool, expression of *ISG15*, *ISG20*, and *OAS1* was quantified by qRT-PCR, and neutral lipid accumulation was assessed by Boron-dipyrromethene (BODIPY) 493/503 staining as described below (**Supplementary Figure 8**). The siRNA catalog identifiers are listed in **Supplementary Table 5**, and target sequences are listed in **Supplementary Table 6**.

For experiments involving sequential siRNA transfections, HepG2 cells were first transfected (reverse) with siRNAs targeting innate immune signaling components: interferon alpha/beta receptor subunit 1 (IFNAR1), TMEM173 (stimulator of interferon genes [STING]), DDX58 (RIG-I; cytosolic RNA sensor), or RELA (p65; NF-κB subunit). After 48 h, cells were retransfected (forward) with siRNA targeting DGUOK, either alone or in combination with each innate immune siRNA, to assess pathway-specific modulation of lipid accumulation. A nontargeting siRNA (siCON) served as a negative control. Cells were collected 72 h post-DGUOK transfection for RNA, protein, and lipid imaging analyses.

### 2.3 Plasmid construct and CRISPR-Cas9 transfection

The plasmid backbone, Pb-513B-1 (System Biosciences Palo Alto, CA, USA), a PiggyBac vector with a blasticidin resistance cassette and green fluorescent protein (GFP) reporter gene, was used to construct single-guide RNA (sgRNA) plasmids targeting DGUOK exons 1, 2, and 3 [sequences: AGGAGGAAACGCCCTCGAGT (−), CTACAGAACCTGTAGCAACA (+), TGTACCGGGAGCCAGCACGA (+)]. Plasmids were generated using NEBuilder HiFi DNA Assembly (New England Biolabs Ipswich, MA, USA) following BbsI digestion, which produced two 5-kb fragments and a ~ 22-bp fragment. Fragments were purified using a gel recovery kit (Qiagen Hilden, Germany), and Gibson assembly was designed to integrate sgRNA sequences into the plasmid and repair the blasticidin cassette cut site. Assembled constructs were transformed into Stbl3 bacteria (Invitrogen Carlsbad, CA, USA), plated on ampicillin plates, and validated by sequencing.

HepG2 cells were seeded at 100,000 cells/well in a 24-well plate and transfected with plasmids targeting DGUOK via electroporation (300 ng plasmid DNA/well with 0.076 µL transposase/well). Electroporation was performed using Buffer R (Invitrogen) and settings of 1230-20–3 with the Neon Transfection System (Invitrogen). All three sgRNA constructs were transfected into the same well to maximize targeting efficiency. Posttransfection, cells underwent selection with 1.5 µg/mL treatment of blasticidin S (Gibco Grand Island, NY, USA) for 6 weeks.

Following antibiotic selection, HepG2 DGUOK KO cells were maintained and analyzed as a polyclonal population rather than isogenic clonal knockout lines. Knockout efficiency in the polyclonal population was assessed by measuring DGUOK expression by qRT-PCR and Western blotting.

### 2.4 Total RNA extraction and qRT-PCR

Total cellular RNA was extracted using the Quick-RNA Miniprep Plus Kit (Zymo Research Irvine, CA, USA), and cDNA was synthesized using the qScript cDNA synthesis kit (Quanta Beverly, MA, USA), per manufacturer’s instructions. qRT-PCR was performed using the CFX96 or CFX384 Touch Detection System (Bio-Rad Hercules, CA, USA), with Taqman Universal PCR Master Mix (Applied Biosystems Waltham, MA, USA). Messenger RNA abundance of each gene was normalized to Beta-actin (ACTB) levels in the Taqman assay (Thermo Fisher Scientific). Primer sequences are listed in **Supplementary Table 1**.

### 2.5 Western blotting

For whole-cell lysates, cells were washed in chilled phosphate-buffered saline (PBS) and lysed in 1× RIPA buffer with Protease/Phosphatase Inhibitor Cocktail (Cell Signaling Technology Danvers, MA, USA). After rotation for 30 min at 4°C, lysates were centrifuged at 15,000 rpm for 15 min to collect supernatants. Protein concentration in supernatants was quantified using Pierce bicinchoninic acid (BCA) Protein Assay Kit (BCA assay, Thermo Fisher Scientific), and 50 μg was loaded per well. Sample preparation, gel electrophoresis, transfer, and blocking were performed as previously described (18). Blots were incubated with DGUOK primary antibody (1:500; Cat. No. sc-398101, Santa Cruz Dallas, TX, USA) at 4°C overnight, with ACTB incubation (1:10,000) at 22°C for 1 h. Blots were washed with 0.1% Tris-buffered saline with Tween 20 and incubated with horseradish peroxidase-conjugated secondary antibodies (1:1,000, Cell Signaling Technology) at 22°C for 1 h. Signal was detected using Enhanced Chemiluminescence (ECL) Reagent (Bio-Rad) and imaged on the Bio-Rad Gel Doc.

For protein-level validation of siRNA knockdowns, whole-cell lysates were analyzed by Western blotting with antibodies against IFNAR1 (83002-4-RR, Proteintech Rosemont, IL, USA), p65/RELA (Santa Cruz Biotechnology, sc-372), STING/TMEM173 (D2P2F, No. 13647, Cell Signaling Technology), RIG-I/DDX58 (ab45428, Abcam), and IFNGR1 (EPR7866, Cat. No. ab134070, Abcam Cambridge, UK), and ACTB was used as a loading control.

### 2.6 mtDNA copy number quantification

Total cellular DNA was extracted using the Quick-DNA Miniprep Plus Kit (Zymo Research). Mitochondrial DNA (mtDNA) copy number was quantified by quantitative PCR (qPCR) on a CFX96 or CFX384 Touch Real-Time PCR Detection System (Bio-Rad) using TaqMan Universal PCR Master Mix (Applied Biosystems). MT-CO1 and MT-ND2 were each normalized to the nuclear gene HBB (Thermo Scientific), and relative copy number was calculated using the 2^–ΔΔ^Ct method (27–29).

### 2.7 Transmission electron microscopy

siCONsiCON and siDGk transfections were carried out for 72 h using standard protocols to ensure efficient knockdown of the target gene. HepG2 WT and KO cells were cultured on 100 mm dishes, with Blasticidin S selection applied for 5 days to ensure the selection of successfully modified cells. Cells were fixed in 2.5% glutaraldehyde prepared in 0.1 M sodium cacodylate buffer and incubated at room temperature for 1 h, followed by 4°C incubation for 96 h. The fixed samples were processed by the University of California San Diego (UCSD) Electron Microscopy (EM) Core Facility. Sample processing involved fixation in glutaraldehyde, incubation in 1% osmium tetroxide, staining with 2% uranyl acetate, dehydration through a graded ethanol series, resin infiltration and polymerization, ultrathin sectioning, and poststaining with 2% uranyl acetate and lead citrate.

Electron microscopy images were analyzed using ImageJ (30, 31) to quantify mitochondrial and lipid droplet morphology (32, 33). Mitochondrial size was defined as the cross-sectional area of individual mitochondria within each cell, while cytoplasmic area was calculated by subtracting the nuclear area from the total cellular area. Lipid droplet accumulation was determined as the total lipid droplet area within the cytoplasm divided by the cytoplasmic area of each cell. Lipid droplet size was defined as the area of individual lipid droplets. Organelle occupancy was expressed as the ratio of organelle area to cytoplasmic area, reported as a percentage.

### 2.8 RNA bulk-sequencing mapping and differential expression analysis

The paired-end reads that passed Illumina filters were filtered for reads aligning to transfer RNA (tRNA), ribosomal RNA (rRNA), adapter sequences, and spike-in controls. The reads were then aligned to the GRCh38 reference genome and Gencode v27 annotations using STAR (v2.6.1) (34). DUST (low-complexity sequence filtering algorithm) scores were calculated with PRINSEQ Lite (v0.20.3), and low-complexity reads (DUST > 4) were removed from the Binary Alignment/Map (BAM) files (34, 35). The alignment results were parsed using SAMtools to generate SAM files. Read counts to each genomic feature were obtained using featureCounts (v1.6.5) using the default parameters along with a minimum quality cutoff (Phred > 10) (36, 37). After removing absent features (zero counts in all samples), the raw counts were then imported to the R/Bioconductor package DESeq2 (v1.24.0) to identify differentially expressed genes among samples. *p*-values for differential expression were calculated using the Wald test for differences between the base means of two conditions (38). These *p*-values were then adjusted for multiple test corrections using the Benjamini–Hochberg algorithm (39). We considered genes differentially expressed between two groups of samples when the DESeq2 analysis resulted in an adjusted *p*-value of < 0.05 and the shrunken |log_2_FC| > 0.5. Principal component analysis (PCA) was performed using the ‘prcomp’ function in R. The sequencing data generated in this article have been submitted to the Gene Expression Omnibus under Accession Number GSE309813 (http://www.ncbi.nlm.nih.gov/geo/).

Volcano and MA plots were generated in ggplot2 v3.5.1. The volcano plot displays log2 fold-change (x) vs. –log10(adjusted *p*-value) (y). Gene set enrichment used GSEA-Preranked (classic scoring) with Gene Ontology (GO) gene sets from Molecular Signatures Database (MSigDB); ranks were –log10(*P*) for positive log_2_FC and +log10(*P*) for negative log2FC. Since GSEA was performed on a ranked list of all detected genes, pathway-level enrichment statistics were not dependent on the adjusted *p*-value threshold used to define differentially expressed genes for volcano plot visualization.

### 2.9 HERV analysis

For analysis of HERV expression, the paired-end reads that passed Illumina filters were filtered for reads aligning to tRNA, rRNA, adapter sequences, and spike-in controls. The reads were then aligned to the reference genome using TopHat (v1.4.1) and alignment results parsed via SAMtools to generate SAM files. The uniquely and multimapped reads were filtered from the BAM files using a mapping quality (MAPQ) of 255 to filter uniquely mapped reads. Read counts to each repeat element were obtained by mapping the multimapped reads to each of the repeat classes and counting alignment for each repeat class (all the steps are part of the RepEnrich pipeline). After removing absent repeat elements (zero counts in all samples), the raw counts were then imported to the R/Bioconductor package EdgeR to identify differentially expressed genes among samples. The generalized linear model method within the EdgeR package was used to identify differentially expressed genes. We considered genes differentially expressed between two groups of samples when the EdgeR analysis resulted in an adjusted *p*-value of < 0.05.

### 2.10 BODIPY lipid staining

#### 2.10.1 Cellular imaging and analysis

The day before staining, 10,000 cells were seeded per well in black, clear-bottom, glass 96-well plates precoated with 0.01 mg/mL poly-d-lysine. The following day, cells were washed once with PBS and fixed with room-temperature 4% paraformaldehyde for 15 min. After fixation, cells were washed three times with PBS and incubated with 2 µM fluorescein isothiocyanate (FITC)-BODIPY 493/503 in PBS for 30 min at room temperature in the dark on a rocker. Excess dye was removed by washing the cells three times with PBS.

Cells were then counterstained with 4′,6-diamidino-2-phenylindole (DAPI) (1:10,000) for 5 min in the dark, followed by three additional PBS washes. Imaging was performed using a Keyence BZ-X series fluorescence microscope. For each well, six fields were randomly imaged, with a minimum of five wells analyzed per condition per experiment.

Images were analyzed using QuPath Software (40). Lipid droplets and nuclei were identified using the subcellular detection tool. Detection parameters were adjusted to exclude objects smaller than 0.2 μm^2^ and minimize background artifacts. Total lipid droplet counts were normalized to the number of DAPI-stained nuclei to calculate the average droplet count per cell. At least five images per well and five wells per condition were analyzed, using only images with comparable confluency to reduce variability. Image analysis was performed blinded to the experimental condition.

#### 2.10.2 Flow cytometry analysis

Following treatment with 48-hour 2′-deoxyadenosine (dAdo; Sigma-Aldrich St. Louis, MO, USA) or 72 h siDGk, cells were detached with 0.25% trypsin (Thermo Scientific), neutralized, and washed with PBS. Cells were incubated with 2 µM BODIPY 493/503 at 37°C for 15 min, protected from light, then washed and resuspended in 200 µL fluorescence-activated cell sorting (FACS) buffer (PBS, 2 mM EDTA, 2% FBS, 0.05% sodium azide) containing propidium iodide (PI; 1:200; BioLegend San Diego, CA, USA). Samples were kept protected from light, on ice, and acquired within 30 min on a BD LSR-II Flow Cytometer. BODIPY fluorescence was collected in the FITC channel; PI was collected in a red PI-compatible channel. Data were analyzed in FlowJo v10 (41). Dead cells (PI^+^) were excluded, and the BODIPY signal was quantified in the live (PI^-^) gate. Unstained and single-stain compensation controls were included (BODIPY-only cells; PI-only cells generated by heat-killing at 56°C for 10 min), and the compensation matrix was calculated in FlowJo and applied to all samples.

### 2.11 Oil Red O staining and quantification

Oil Red O staining was performed using the Sigma-Aldrich Lipid (Oil Red O) Staining Kit (Sigma-Aldrich), following the manufacturer’s instructions with modifications described below, to optimize staining for HepG2 cells.

All steps were performed at 22°C unless otherwise specified. Cells were fixed in 10% paraformaldehyde (PFA) and incubated for 5 min. The PFA was then replaced with fresh 10% PFA, and the cells were incubated for 15 min at room temperature on a rocker. Following fixation, the cells were washed with 60% isopropanol and incubated for 1 min. The cells were allowed to air dry for 10 min before the addition of the Oil Red O (ORO) working solution. The cells were then incubated with the ORO working solution for 10 min at room temperature on a rocker. After incubation, the cells were washed five times with distilled water, covered with water, and imaged using a light microscope.

For quantification, water was removed from the wells, and cells were air-dried in a sterile hood for 10 m. Isopropanol at 100% was then added to each well to elute the ORO stain, followed by a 10-m incubation at room temperature on a rocker. The solution was mixed thoroughly, transferred to a fresh 96-well plate, and absorbance was measured at 540 nm with background correction at 620 nm using a Spectramax plate reader (Molecular Sciences San Jose, CA, USA).

### 2.12 IFN-β neutralization assay

Recombinant human IFN-β neutralizing antibody (nAb; R&D Systems Minneapolis, MN, USA) was added immediately following siRNA transfection at a final concentration of 40 U/mL. Cells were incubated for 72 h posttransfection before RNA isolation, qRT-PCR, and BODIPY lipid staining analyses. Effective IFN-β neutralization was verified by reduced ISG15 messenger RNA (mRNA) expression as measured by qRT-PCR.

### 2.13 JAKi treatment

For pharmacologic inhibition of Janus kinase/signal transducer and activator of transcription (JAK/STAT) signaling, HepG2 cells were treated with ruxolitinib (tlrl-rux-3, InvivoGen San Diego, CA, USA), baricitinib (7222, Tocris Bristol, UK), or upadacitinib (7783, Tocris Bristol, UK). JAK inhibitors (JAKi) concentrations were based upon ruxolitinib titration experiments measuring inhibition of IFN-γ-induced STAT1 phosphorylation, assessed by Western blotting with phospho-STAT1 Tyr701 antibody (9167T, Cell Signaling Technology) and ACTB as a loading control (**Supplementary Figure 10**). For lipid accumulation experiments, assessed by BODIPY 493/503 staining, HepG2 cells were transfected with siCONsiCON or siDGk for 48 h and treated with 5, 10, or 50 nM JAKi (IC_50_ = 2.8–43 nM) for 24 h prior to harvest. Protein lysates and lipid staining samples were collected at 72 h posttransfection. Baseline lipid accumulation in siCON cells was assessed following JAK inhibitor treatment, and no increase in lipid droplet accumulation was observed under these conditions (**Supplementary Figure 10**). Recombinant human IFN-γ (100 U/mL for 24 h; 285-IF-025, R&D Systems) was used as a positive control for STAT1 phosphorylation.

### 2.14 2′-Deoxyadenosine treatment

dAdo (Sigma-Aldrich) was reconstituted to 80 mM in sterile DNase-/RNase-free water. To determine effective dAdo concentrations for downstream experiments, cell viability following dAdo treatment was assessed by caspase-3/7 activation and SYTOX exclusion. Wild-type HepG2 cells were seeded at 2.5 × 10^5^ cells per well in 6-well plates and allowed to adhere overnight. The following day, cells were treated with 0, 100, 250, 500, 750, 1000, or 1,500 µM dAdo in EMEM supplemented with 10% FBS for 48 h. After treatment, cells were collected, transferred to a 96-well plate, washed twice with FACS buffer, and pelleted. Cell pellets were resuspended in 100 µL Caspase-3/7 Detection Reagent (Cat. No. C10740, Thermo Fisher Scientific) diluted 1:1,000 in prewarmed FACS buffer and incubated at 37 °C for 25 min according to the manufacturer’s instructions. SYTOX solution was then added at a final dilution of 1:500, and samples were incubated for an additional 5 min at 37 °C. Samples were analyzed on a BD LSR II flow cytometer using BD FACSDiva software. Staurosporine treatment (2 µM, 24 h) was included as a positive control for apoptosis induction. Gating strategy is provided in **Supplementary Figure 12**.

dAdo concentrations that did not significantly alter cell viability were selected for downstream lipid, methylation, and gene expression analyses. HepG2 cells were seeded into 6- or 24-well plates with 2.0 or 0.5 mL medium per well, respectively. At 60%–70% confluency, cells were treated with 0, 250, 500, or 1,000 µM dAdo in EMEM supplemented with 10% FBS. Treatments were prepared from the 80 mM stock and vehicle-matched across conditions. Cells were maintained at 37 °C with 5% CO_2_ and harvested at 24 h for methylation analysis or 48 h for RT-qPCR and flow cytometry-based BODIPY lipid analysis. For dAdo pathway-intervention experiments, HepG2 cells were first transfected with IFNAR1 siRNA, then treated with dAdo at 750 µM for 48 h prior to fixation and BODIPY 493/503 staining. Lipid accumulation was quantified by fluorescence imaging as described below. Knockdown validation for all pathway-targeting siRNAs was performed by Western blotting, as described above.

### 2.15 Global DNA methylation (5-mC) ELISA

After 48 h of siRNA transfection or 24 h of dAdo treatment, DNA was isolated from cells using the DNeasy Blood and Tissue Kit (Qiagen). Global 5-methylcytosine (5-mC) was quantified using the 5-mC DNA ELISA Kit (Zymo Research) with 100 ng genomic DNA per well. DNA was denatured at 98°C for 5 min, chilled on ice for 10 min, and the full volume was added to the ELISA plate in a coating buffer (final 100 µL). A 0%–25% 5-mC standard curve was run in duplicate on each plate. Absorbance was read at 450 nm. Standard curves were fitted with a four-parameter logistic (4PL; untransformed X) model using 1/Y^2^ weighting, and sample values were interpolated from the fitted curve. Standards/samples outside the calibrated range were excluded or reassayed after dilution. Reported values are the mean of technical duplicates; where indicated, data are shown as fold-change vs. control siRNA (siCON).

### 2.16 Bisulfite conversion of DNA

Genomic DNA was isolated from HepG2 cell cultures using the DNA Clean and Concentrator-5 Kit (No. D4014, Zymo Research) according to the manufacturer’s instructions. DNA concentration and purity were assessed by NanoDrop spectrophotometry prior to bisulfite conversion to confirm sufficient yield and quality (A260/A280 ≥ 1.8). Five hundred nanograms of genomic DNA were subjected to bisulfite modification using the EZ DNA Methylation-Gold Kit (No. D5006, Zymo Research) per the manufacturer’s protocol. In this reaction, sodium bisulfite deaminates all unmethylated cytosines (uC) to uracil, which is subsequently read as thymine (T) during PCR amplification, while methylated cytosines (mC) are protected from conversion and are retained as cytosine. Bisulfite-converted DNA was eluted and stored at − 20°C until further use.

### 2.17 Primer design and methylation-sensitive high-resolution melting analysis

#### 2.17.1 CpG island identification and primer design

Cytosine-phosphate-guanine (CpG) islands within the regulatory and promoter-proximal elements of the ISG15 and ISG20 genomic loci were identified using the UCSC Genome Browser (https://genome.ucsc.edu/). Promoter-associated sequences were retrieved and submitted to MethPrimer 2 (https://www.urogene.org/methprimer2/) for CpG island prediction and bisulfite-specific PCR (BSP) primer design. Primers were designed to amplify amplicons of 80–250 bp spanning the identified CpG islands and were synthesized by Integrated DNA Technologies (IDT). Primer sequences were as follows: ISG15 forward, 5′-GTATTTTGTGAAGGATTTGGAATG-3′ and reverse, 5′AATAACCAAATTTAACTTCAATTTC3′ (product size = 200 bp); ISG20 forward, 5′-TAGTTTTGGGGATGTTTATTTTTTG-3′ and reverse, 5′-CCATATCAAAATACCCCTCCTTTA-3′ (product size = 134 bp).

#### 2.17.2 Bisulfite-specific PCR

PCR amplification of bisulfite-converted DNA was performed using TaKaRa EpiTaq HS polymerase (No. R110B, TaKaRa Bio Shiga, Japan), which is specifically formulated for bisulfite-treated templates. Each 50 µL reaction contained 5 µL of 10× EpiTaq PCR buffer, 2.5 mM MgCl_2_, 0.2 mM each dNTP, approximately 100 ng of bisulfite-converted DNA, 400 nM of each forward and reverse primer, and 0.25 µL of EpiTaq HS polymerase, brought to volume with nuclease-free water. Thermal cycling was performed on a T100 Thermal Cycler (Bio-Rad) with the following program: initial denaturation at 98 °C for 2 min; 40 cycles of 94 °C for 20 s, 55 °C for 30 s, and 72 °C for 30 s; and a final extension at 72 °C for 10 min. Successful amplification was confirmed by electrophoresis on a 1.5% agarose gel (**Supplementary Figure 13**). PCR products were purified using the QIAquick PCR Purification Kit (No. 28106, Qiagen) and eluted in 50 µL of nuclease-free water.

#### 2.17.3 High-resolution melting analysis

Two microliters of purified PCR product were used as a template in the high-resolution melting (HRM) qPCR reaction. Reactions were prepared using Precision Melt Supermix for HRM Analysis (No. 1725110, Bio-Rad) according to the manufacturer’s instructions, using the same BSP primers at 400 nM each. HRM qPCR was performed on a CFX384 Touch Real-Time PCR Detection System (Bio-Rad) with the following program: initial denaturation at 95 °C for 2 min; 40 cycles of 95 °C for 10 s and 55 °C for 30 s; followed by a high-resolution melting step consisting of 95 °C for 30 s, 60 °C for 1 min, and a continuous melt ramp from 65 °C to 95 °C in 0.2 °C increments with a 10-s hold per step. The resulting negative derivative of fluorescence with respect to temperature (−d*F*/d*T*, RFU/°C) melt curves were analyzed to assess methylation-dependent differences in amplicon melting profiles across treatment groups.

### 2.18 Statistical analysis

Statistical analyses were performed using Excel (Microsoft 2019) and GraphPad Prism version 9.4.1, GraphPad Software (San Diego, CA, USA). At least two independent experiments were performed, and error bars represent the mean ± standard error of the mean. Two-group comparisons used unpaired, two-tailed Student’s *t*-tests. For comparisons across ≥ 3 groups, one-way analysis of variance (ANOVA) with Dunnett’s multiple-comparisons test was used to compare each treatment to the baseline control. Exact *p-*values are reported in the figure legends.

For methylation-sensitive high-resolution melting (MS-HRM) data, peak −d*F*/d*T* amplitudes were compared across siRNA treatment groups (siCONsiCON, siDGksiDGk, and siDNMT1) at each time point and gene independently. Prior to group comparisons, normality was assessed using the Shapiro–Wilk test, and variance homogeneity was evaluated using Levene’s test. As data across conditions violated the assumptions of normality and homoscedasticity, nonparametric Kruskal–Wallis tests were used to assess overall group differences, followed by Dunn’s multiple comparisons test with Bonferroni correction. All MS-HRM statistical analyses were performed in GraphPad Prism.

## 3 Results

### 3.1 Mitochondrial structure and function are intact in early DGUOK deficiency

#### 3.1.1 mtDNA copy number

**Figure 1:**
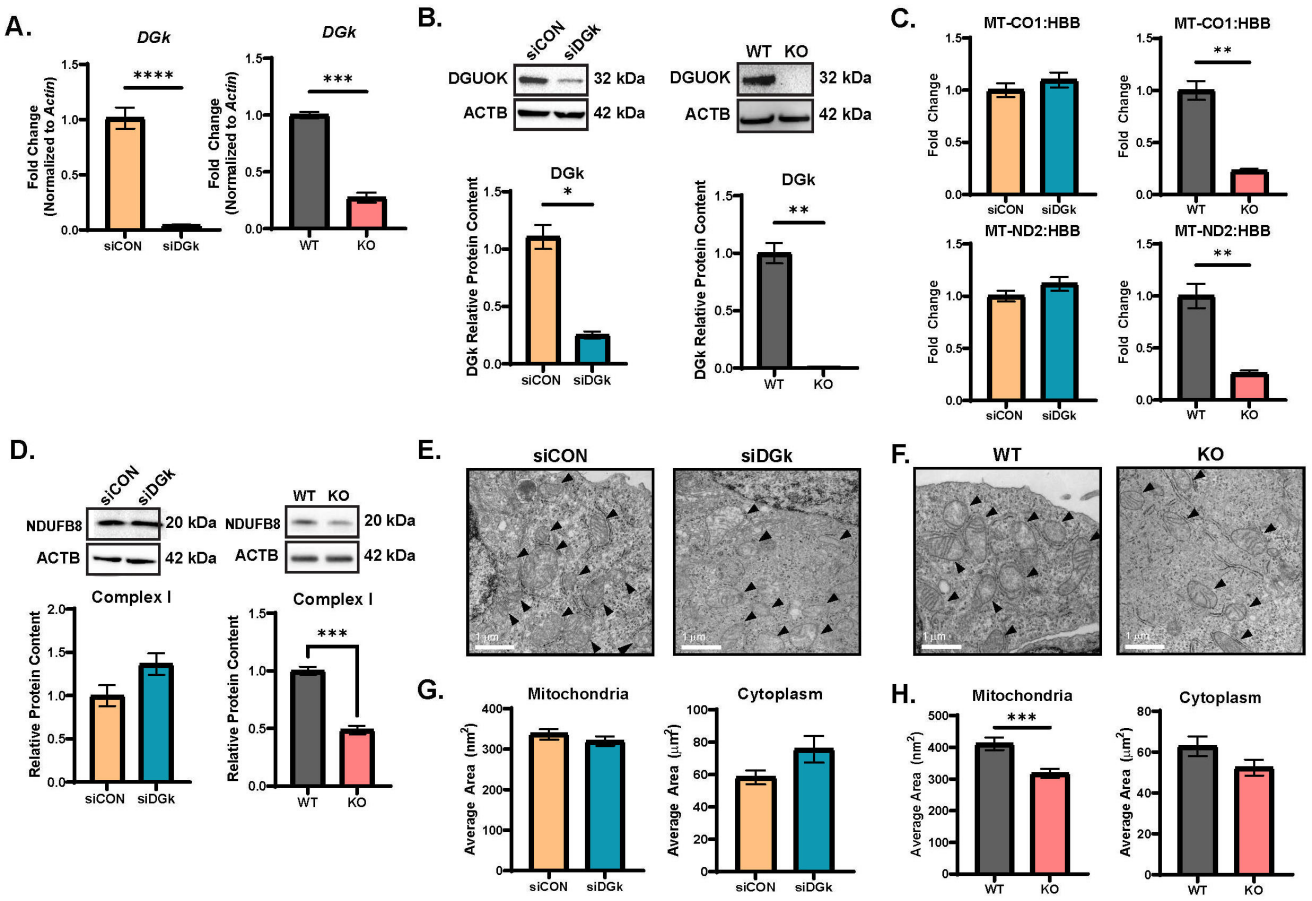
Transient DGUOK knockdown maintains mitochondrial DNA (mtDNA) integrity and preserves mitochondrial morphology. **(A)** DGUOK mRNA expression was quantified by RT-qPCR in transient siRNA knockdown (siDGk) and CRISPR-Cas9 knockout (KO) HepG2 cells compared with their respective controls (siCON, WT). DGUOK expression was reduced by 97% in siDGk (^****^p = 0.0004; *n* = 3) and by ~ 90% in KO (^***^p < 0.001, *n* = 3) **(B)** DGUOK protein levels were assessed by Western blot at 72 h posttransfection or after chronic KO establishment, showing ~ 75% loss in siDGk (*p = 0.0457, *n* = 2) and ~ 85% loss in KO (**p = 0.0082, *n* = 3). **(C)** mtDNA copy number was determined by qPCR using MT-CO1:HBB and MT-ND2:HBB ratios. Transient knockdown showed no significant change (siCON vs. siDGk: MT-ND2 *p* = 0.2986, MT-CO1 *p* = 0.4273, *n* = 2–3), whereas KO cells exhibited significant mtDNA depletion (WT vs. KO: MT-ND2 *p* = 0.0034, MT-CO1 *p* = 0.0012, *n* = 3). **(D)** OXPHOS complex analysis showed a 51% reduction in complex I in KO cells (WT = 1.00, KO = 0.4878, *p* = 0.006, *n* = 3), with no changes in complexes II–V (*p* > 0.2). siDGk cells maintained normal complex I levels (*p* = 0.17, *n* = 2) (full OXPHOS panel shown in **Supplementary Figure 1**). **(E–H)** Transmission electron microscopy (TEM) revealed normal mitochondria (arrowheads) in siDGk cells **(E**, **G)**, but a 22.6% reduction in mitochondrial cross-sectional area in KO cells [**(F**, **H)**; WT = 411.0 nm^2^, KO = 318.2 nm², *p* = 0.0001, *n* = 259–336 mitochondria from 30 cells per group]. Cytoplasmic area and mitochondrial occupancy were unchanged between groups (*p* > 0.05) (additional quantifications are provided in **Supplementary Figure 2**). All data represent mean ± SEM; significance was determined using unpaired two-tailed *t*-tests (*p < 0.05, **p < 0.01, ***p < 0.001, ****p < 0.0001).

To define the early consequences of DGUOK loss in hepatocytes, siRNA-mediated silencing of DGUOK (siDGk) was performed in HepG2 cells. Knockdown efficiency was confirmed by an observed 97% reduction in *DGUOK* mRNA at 48 h and > 75% reduction of DGUOK protein at 72 h relative to the control nontargeting siRNA (siCON) (**Figures 1A, B**). Despite robust DGUOK depletion, mtDNA copy number quantified by qRT-PCR as Mitochondrially Encoded NADH: Ubiquinone Oxidoreductase Core Subunits 1 and 2 (MT-ND1 and MT-CO1) abundance normalized to Hemoglobin Subunit Beta (HBB) (27–29), remained unchanged 96 h posttransfection (**Figure 1C**). In contrast, CRISPR/Cas9-mediated DGUOK knockout (KO) cells examined after six weeks in culture exhibited > 75% decrease in mtDNA copy number, confirming that prolonged, but not acute, loss of DGUOK expression is required for mtDNA depletion (*p* = 0.0034; **Figure 1C**).

#### 3.1.2 Mitochondrial function and morphology

Given that mtDNA levels remained stable following transient DGUOK knockdown, we assessed whether functional or structural mitochondrial defects emerged prior to mtDNA depletion. Western blotting analysis of respiratory chain complexes revealed that siDGk cells maintained normal complex I levels, whereas KO cells exhibited a ~ 50% reduction in complex I protein, consistent with impaired assembly of mtDNA-encoded subunits (9, 42) (*p* = 0.006; **Figure 1D**; **Supplementary Figure 1**). Transmission electron microscopy further demonstrated preserved mitochondrial morphology in siDGk cells, while KO cells showed a 22.6% reduction in mitochondrial cross-sectional area, consistent with ultrastructural stress and fragmentation associated with mtDNA maintenance disorders (43) (*p* = 0.0001; **Figures 1E–H**). Cytoplasmic area and mitochondrial occupancy were unchanged (**Supplementary Figure 2**). Collectively, these findings indicate that mitochondrial respiration and organelle structure are maintained during early DGUOK depletion and that mitochondrial defects arise only after prolonged loss of DGUOK.

### 3.2 Early DGUOK deficiency reprograms immune, lipid, and methylation/nucleotide pathways

**Figure 2:**
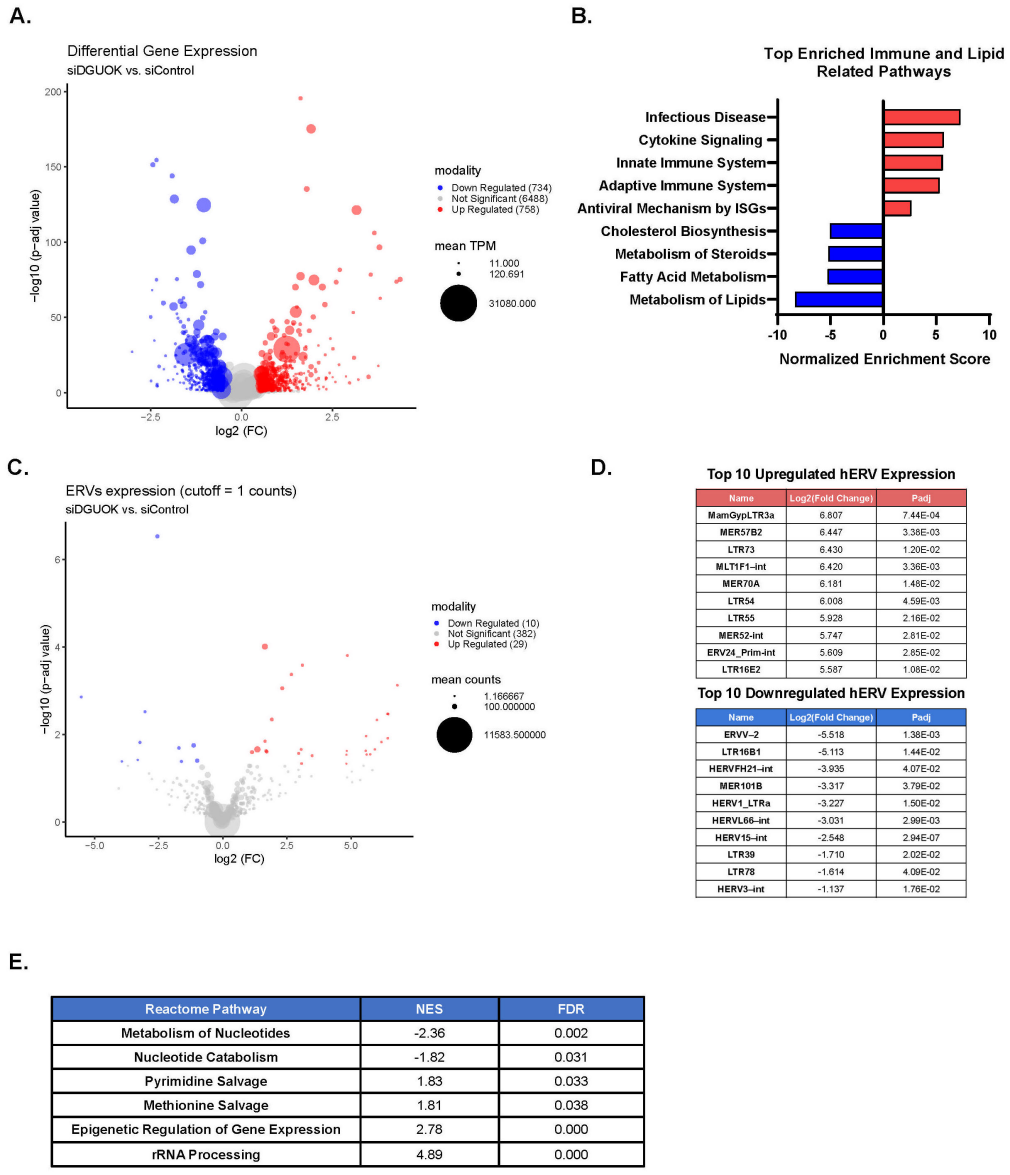
RNA bulk sequencing reveals positive enrichment in innate immune pathways and altered lipid metabolism in DGUOK knockdown cells. **(A)** Volcano plot of differentially expressed genes (DEGs) between siDGk (siDGk) and siCON HepG2 cells from bulk RNA-seq analysis (DESeq2, *n* = 3 per group). Differential expression was defined using adjusted p-value ≤ 0.05, shrunken |log_2_FC| > 0.5, and mean TPM > 10. A total of 1,492 genes were differentially expressed, including 758 upregulated and 734 downregulated genes. **(B)** Gene set enrichment analysis (GSEA, Reactome) revealed strong upregulation of immune and antiviral pathways, including interferon signaling (NES = 2.81, FDR = 6.9 × 10^−5^), antiviral mechanisms (NES = 2.68, FDR = 2.0 × 10^−4^), and cytokine signaling (NES = 5.69, FDR < 0.001). Lipid metabolism pathways were conversely suppressed, including metabolism of lipids (NES = − 8.34) and fatty acid metabolism (NES = − 5.26). **(C, D)** EdgeR analysis of human endogenous retrovirus (hERV) elements identified 39 significantly altered loci (padj < 0.10), including upregulated members of the ERVK, HERVH, and HERVW families linked to interferon-responsive programs. **(E)** Additional GSEA enrichment included nucleotide- and methylation-associated pathways: metabolism of nucleotides, methionine salvage, and epigenetic regulation of gene expression.

To define transcriptional and signaling responses that precede overt mitochondrial dysfunction, siRNA-mediated DGUOK depletion was performed prior to bulk RNA sequencing. This temporally controlled approach enabled assessment of early changes captured before mtDNA depletion, respiratory chain defects, or long-term compensatory remodeling. Differential expression analysis identified 1,492 significantly altered genes (adjusted *p*-value ≤ 0.05, shrunken |log_2_FC| > 0.5, and mean TPM > 10) (**Figure 2A**; **Supplementary Figure 3**). Gene set enrichment analysis using the Reactome database revealed two major categories of transcriptional remodeling: immune signaling pathways were positively enriched in siDGk cells, whereas lipid metabolism pathways were negatively enriched (**Figure 2B**; **Supplementary Figure 4–S6**; **Supplementary Tables 1–S3**). Strong upregulation of innate immune and antiviral pathways included IFN signaling (normalized enrichment score (NES) = 2.81, false discovery rate (FDR) = 6.9 × 10^−5^), antiviral mechanisms by ISG (NES = 2.68, FDR = 2.0 × 10^−4^), cytokine signaling (NES = 5.69, FDR < 0.001), JAK–STAT signaling (NES = 2.25, FDR = 0.004), and viral infection pathways (NES = 6.86, FDR < 0.001) (**Figure 2B**; **Supplementary Figure 4–S6**; **Supplementary Table 4**). Lipid metabolism pathways showed coordinated transcriptional suppression, including metabolism of lipids (NES = − 8.34, FDR < 0.001), fatty acid metabolism (NES = − 5.26, FDR < 0.001), steroid metabolism (NES = − 5.19, FDR < 0.001), and cholesterol biosynthesis (NES = − 5.04, FDR < 0.001) (**Supplementary Table 4I**). Empirical analysis of digital gene expression data in R (EdgeR) of repeat elements identified 39 differentially expressed HERV loci, primarily ERVK, HERVH, and HERVW families, consistent with activation of viral mimicry pathways (18, 44) (**Figures 2C, D**). Mitochondrial-encoded transcripts were largely unchanged aside from modest MT-CO1 and MT-TL1 reduction in siDGk cells, consistent with preserved OXPHOS protein expression and mitochondrial integrity (**Supplementary Table 2**; **Supplementary Figure 7**).

Pathway enrichment further highlighted coordinated perturbations in nucleotide and methyl-donor metabolism (**Figure 2E**), supporting a model in which DGUOK loss perturbs both purine and methionine metabolic networks. Significantly enriched pathways included metabolism of nucleotides (NES = − 2.36, FDR = 0.002), nucleotide catabolism (NES = − 1.82, FDR = 0.031), pyrimidine salvage (NES = 1.83, FDR = 0.033), methionine salvage (NES = 1.81, FDR = 0.038), epigenetic regulation of gene expression (NES = 2.78, FDR < 0.001), and rRNA processing (NES = 4.89, FDR < 0.001). Within these enriched pathways, several genes encoding metabolic enzymes central to nucleotide turnover and methyl-donor metabolism showed altered expression, including 5′-nucleotidase, cytosolic II (NT5C2) (log_2_FC = − 0.58, padj = 1.5 × 10^−4^), PNP (log_2_FC = 1.20, padj = 1.4 × 10^−17^), methionine adenosyltransferase 1A (MAT1A) (log_2_FC = − 0.78, padj = 7.3 × 10^−15^), and adenosylhomocysteinase (AHCY) (log_2_FC = 0.32, padj = 1.7 × 10^−5^). Together, these findings reveal an early and coordinated transcriptional program linking DGUOK loss to innate immune activation, lipid metabolic repression, and altered purine and methyl-donor pathways with minimal effects on mitochondria-encoded gene expression.

### 3.3 DGUOK deficiency induces increased lipid accumulation independently of mitochondrial dysfunction

**Figure 3:**
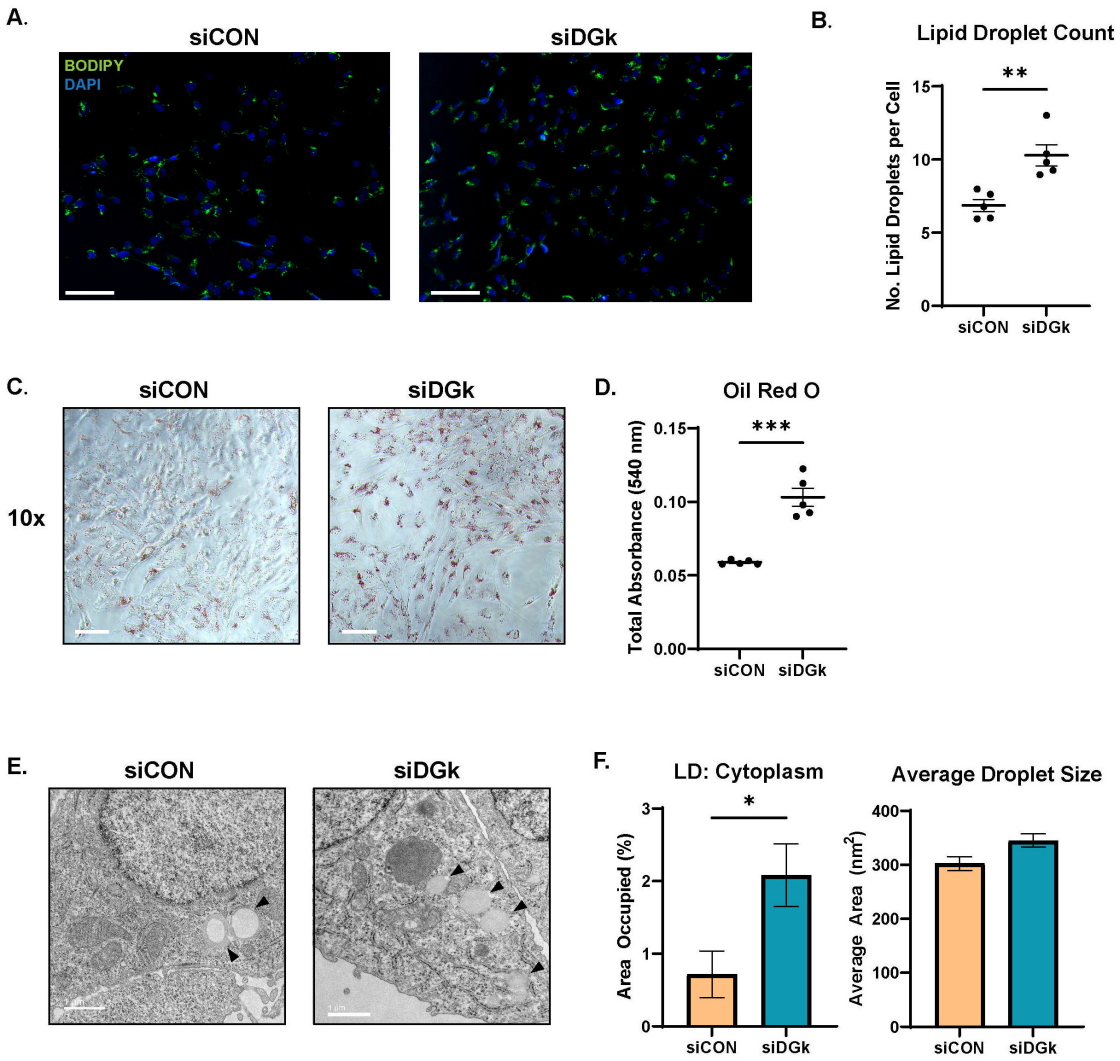
Transient DGUOK knockdown increases lipid accumulation in HepG2 cells. **(A)** Neutral lipid staining using BODIPY 493/503 to visualize lipid droplets in HepG2 cells 72 h posttransfection with siCON or siDGk. Scale bar = 200 µm. **(B)** Quantification of BODIPY staining reveals a significant increase in lipid droplet accumulation in siDGk cells compared with siCON (*p* = 0.0034). Quantification was performed across *n* = 5 fields of view per condition from three independent wells. A minimum of 300 cells were analyzed per condition. **(C)** Oil Red O staining of neutral lipids in siCON and siDGk cells 72 h posttransfection. Scale bar = 100 µm. **(D)** Quantification of Oil Red O staining shows increased lipid accumulation in siDGk cells compared with siCON (*p* = 0.0001). Quantification was performed across *n* = 5 wells per condition. **(E)** Representative transmission electron microscopy (TEM) images showing lipid droplets (arrowheads) in siCON and siDGk HepG2 cells. **(F)** TEM quantification reveals significantly increased numbers of cytoplasmic lipid droplets in siDGk cells, with no change in average droplet size (LD: cytoplasm cell count: *n* = 30 cells, *p* = 0.0153; average droplet size droplet count: siCON *n* = 313, siDGk *n* = 344, *p* = 0.5921). Data are presented as mean ± SEM. Statistical significance was determined using unpaired two-tailed *t*-tests. ns, not significant. For all panels, *p<0.05;**p<0.01;***p<0.001.

Microvesicular and macrovesicular steatosis are frequently reported in the liver of patients with DGUOK deficiency and accompany progressive liver failure (10, 45, 46). Given the broad suppression of lipid metabolism pathways in siDGk cells, we assessed whether DGUOK deficiency promotes steatosis prior to a decline in mtDNA content. 4-Difluoro-4-bora-3a,4a-diaza-s-indacene (BODIPY 493/503) staining of neutral lipids, which are the core components of lipid droplets, revealed that siDGk cells displayed a significant increase in lipid droplet number, averaging 10.29 droplets per cell compared to 6.8 droplets per cell in siCON cells (*p* = 0.0034; **Figures 3A, B**). Oil Red O staining likewise demonstrated a 1.75-fold increase in neutral lipid content (*p* = 0.0001; **Figures 3C, D**). Ultrastructural analysis of lipid morphology was further evaluated using transmission electron microscopy, confirming that siDGk cells exhibited a significant 2.9-fold increase in lipid droplet abundance (*p* = 0.0153; **Figures 3E, F**) with mean droplet size modestly elevated (*p* = 0.0167). These changes occurred despite preserved mitochondrial structure and function, indicating that DGUOK deficiency drives hepatocellular lipid accumulation independent of mtDNA depletion or respiratory chain impairment.

To determine whether the lipid and immune phenotypes observed following siDGk transfection were specific to DGUOK depletion rather than an off-target effect of the pooled siRNA reagent, we next tested two individual, sequence-independent DGUOK-targeting siRNAs. Both siDGk1 and siDGk2 significantly reduced *DGUOK* mRNA (one-way ANOVA, *p* = 0.0021) and DGUOK protein (one-way ANOVA, *p* < 0.0001) expression compared with siCON (**Supplementary Figures 8A, B**). Importantly, each siRNA significantly increased expression of *ISG15*, *ISG20*, and *OAS1* (**Supplementary Figure 8C**). Lipid droplet accumulation was also significantly increased following transfection with siDGk1 or siDGk2 (one-way ANOVA, *p* < 0.0001; **Supplementary Figures 8D, E**). These findings demonstrate that independent DGUOK-targeting siRNAs reproduce the immune and lipid phenotypes observed with the pooled siDGk reagent, supporting the specificity of the DGUOK knockdown phenotype.

### 3.4 Interferon pathway blockade does not reverse siDGk-induced lipid accumulation

**Figure 4:**
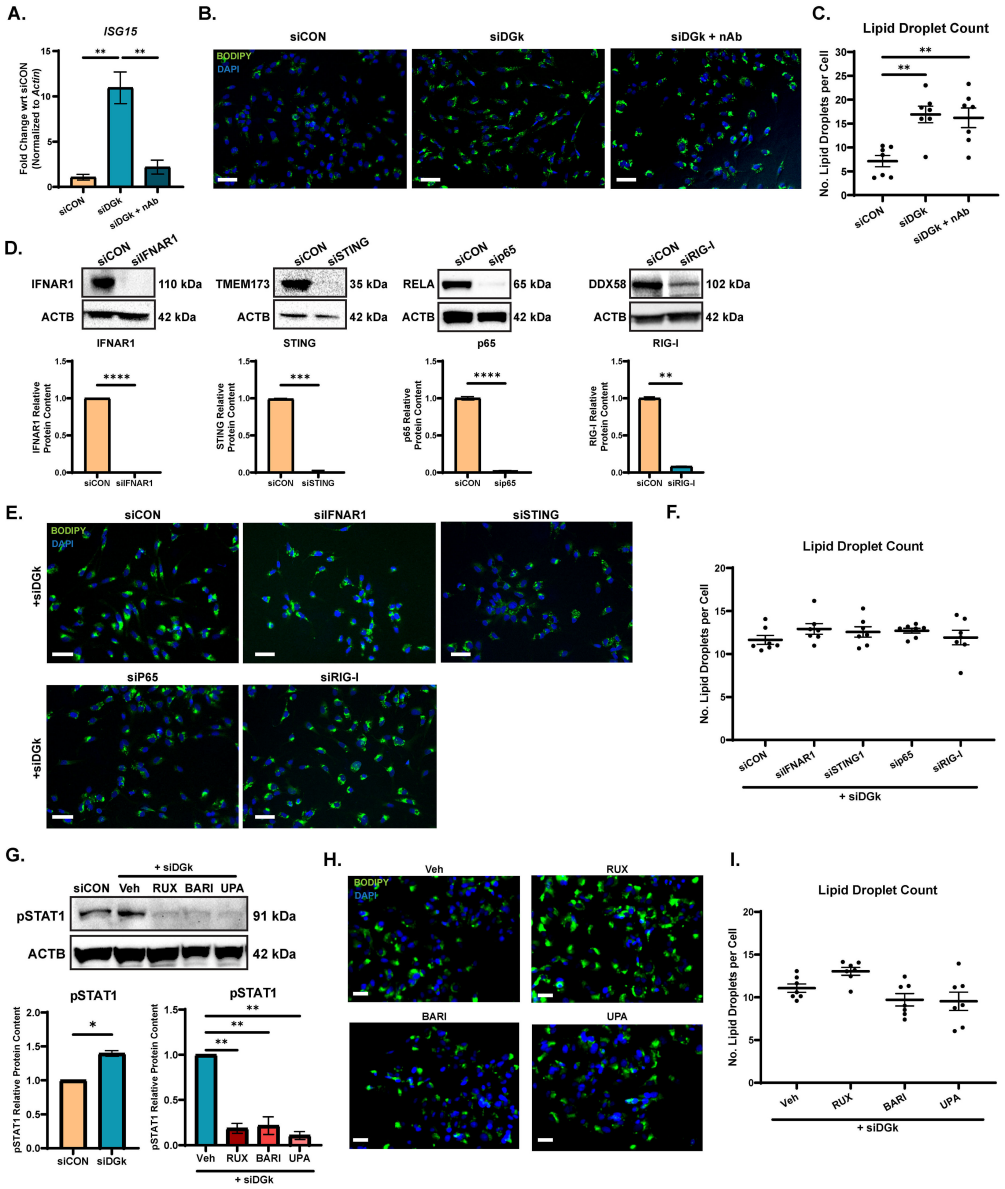
Interferon and innate immune pathway blockade do not reverse siDGk-induced lipid accumulation. **(A)** ISG15 mRNA expression in HepG2 cells transfected with siCON, siDGk (siDGk), or siDGk + IFN-β neutralizing antibody (nAb) (RT-qPCR, *n* = 3; normalized to ACTB; unpaired two-tailed *t*-test, *p* = 0.0051) **(B)** Representative BODIPY 493/503 staining of neutral lipids (green) and DAPI nuclei (blue) 72 h posttransfection. Scale bar = 100 µm. **(C)** Quantification of lipid droplets per cell showing increased lipid accumulation in siDGksiDGk and siDGksiDGk + nAb vs. siCON (one-way ANOVA with Tukey’s multiple comparisons, *n* = 7 wells per condition; mean ± SEM). **(D)** Sequential knockdown validation of IFNAR1, STING, p65, and RIG-I by Western blot (cells pretransfected with innate immune siRNA for 48 h followed by DGUOK silencing for 48 h; *n* = 2; normalized to ACTB; unpaired two-tailed *t*-tests). **(E)** Representative BODIPY/DAPI images of HepG2 cells sequentially transfected with DGUOK and the indicated innate immune siRNAs. Scale bar = 100 µm. **(F)** Quantification of lipid droplets per cell showing that presilencing IFNAR1, STING, p65, or RIG-I prior to DGUOK depletion does not alter lipid accumulation (one-way ANOVA with Tukey’s multiple comparisons, *n* = 7 wells per condition; mean ± SEM). *p* < 0.05; *p* < 0.01; *p* < 0.001; *p* < 0.0001. **(G)** Western blot analysis of phosphorylated STAT1 (pSTAT1) in HepG2 cells transfected with siCON or siDGk and treated with vehicle, ruxolitinib (RUX), baricitinib (BARI), or upadacitinib (UPA). ACTB was used as a loading control. Quantification shows increased pSTAT1 following DGUOK silencing and suppression of pSTAT1 following JAK inhibitor treatment (*n* = 2; mean ± SEM; statistical analysis was performed using an unpaired two-tailed *t*-test for siCON vs. siDGk, and one-way ANOVA with Dunnett’s multiple comparisons test for inhibitor-treated conditions compared with vehicle). **(H)** Representative BODIPY 493/503 staining of neutral lipids (green) and DAPI nuclei (blue) of siDGk-transfected HepG2 cells treated with vehicle, 5 nM RUX, 5 nM BARI, or 5 nM UPA. Scale bar = 100 µm. **(I)** Quantification of lipid droplets per cell from **(H)**, showing that pharmacologic inhibition of JAK/STAT signaling does not reverse siDGk-induced lipid accumulation (*n* = 7 wells per condition; mean ± SEM; one-way ANOVA with Tukey’s multiple comparisons). For all panels, ^*^p < 0.05; ^**^p < 0.01; ^***^p < 0.001; ^****^p < 0.0001. ns, not significant.

Given that type I interferon (IFN-I) signaling has been reported to influence hepatic lipid metabolism, promoting triglyceride accumulation via ISG-dependent remodeling of lipid synthesis and storage (47), we asked whether the steatotic phenotype in DGUOK-deficient cells is driven by interferon activation. Neutralization of IFN-β using monoclonal antibodies (nAb) effectively reduced *ISG15* expression in siDGk cells (*p* = 0.0051; **Figure 4A**), confirming blockade of IFN-I signaling. However, lipid droplet accumulation was not significantly attenuated by IFN-β neutralization (**Figures 4B, C**). To further study IFN-pathway involvement, we performed sequential siRNA silencing of the interferon alpha/beta receptor subunit 1 (IFNAR1), stimulator of interferon genes (STING), NF-κB subunit p65 (RELA), and retinoic acid–inducible gene I (RIG-I, DDX58) prior to DGUOK knockdown. Efficient depletion of each target was confirmed by qRT-PCR, with reductions ranging from 72–88% relative to siCON (*p* < 0.01 for all; **Supplementary Figure 9**), and by Western blotting, with reductions showing > 95% loss of protein (**Figure 4D**). None of these interventions reduced lipid accumulation (**Figures 4E, F**), with mean droplet number remaining unchanged relative to DGUOK knockdown alone. These data demonstrate that while loss of DGUOK induces robust IFN-I signaling, steatosis is independent of innate immune activation.

To further investigate the role of IFN signaling, we tested whether pharmacologic blockade alters the lipid phenotype. Treatment of siDGk cells with the JAKi ruxolitinib (JAK1/2), baricitinib (JAK1/2), and upadacitinib (JAK1) effectively inhibited basal and IFN-γ-induced STAT1 phosphorylation at concentrations near their IC50 values (**Figure 4G**; **Supplementary Figure 10A**). Despite potent JAK/STAT pathway inhibition, siDGk-induced lipid droplet accumulation was not significantly reduced (**Figures 4H, I**). Additional testing across multiple JAKi concentrations similarly showed no reduction in lipid accumulation (**Supplementary Figure 11A**). Finally, silencing IFNGR1 did not significantly alter lipid accumulation in siDGk cells (**Supplementary Figures 11B, C**). Together, data demonstrate that although DGUOK deficiency activates IFN/JAK–STAT signaling, siDGk-induced lipid accumulation is not reversed by genetic or pharmacologic inhibition of IFN signaling or innate immune activation.

### 3.5 Purine supplementation recapitulates lipid and immune phenotypes of DGUOK deficiency

**Figure 5:**
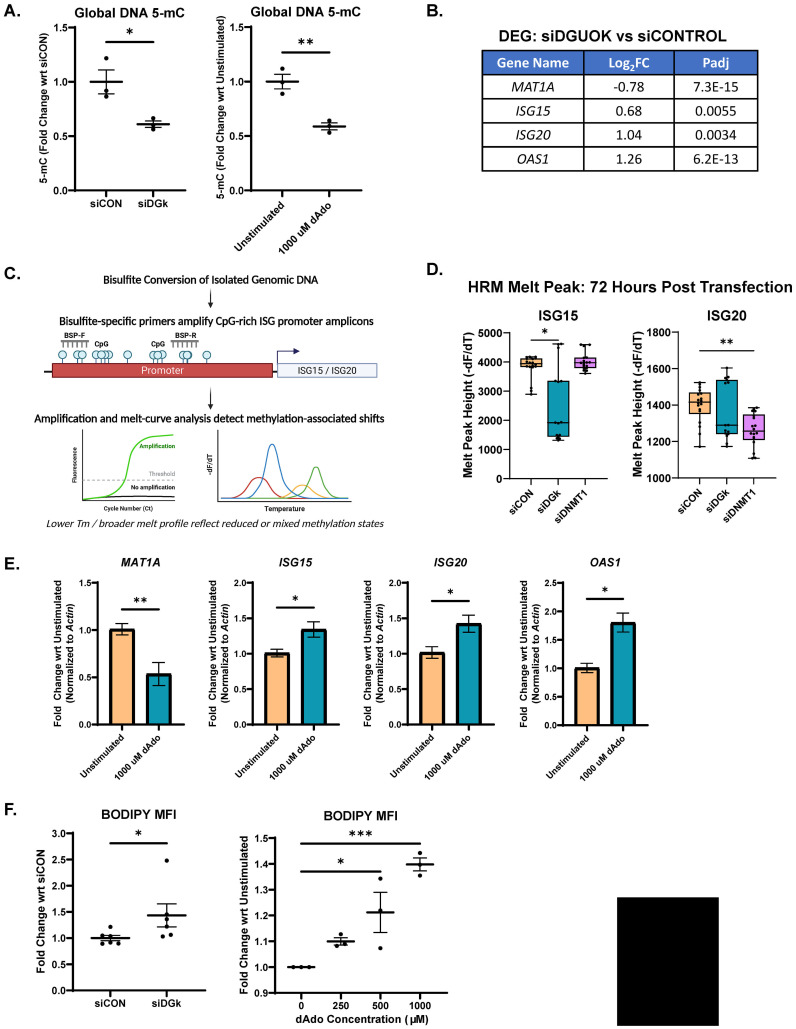
DGUOK depletion and deoxyadenosine exposure link reduced DNA methylation to interferon-stimulated gene expression and lipid accumulation. **(A)** Global DNA 5-methylcytosine (5-mC) quantification by ELISA showing reduced DNA methylation in siDGk (siDGk)-transfected HepG2 cells compared with siCON (unpaired two-tailed *t*-test, *p* = 0.0264, *n* = 3) and in cells treated with 1,000 µM deoxyadenosine (dAdo) compared with unstimulated controls (unpaired two-tailed *t*-test, *p* = 0.0051, *n* = 3). **(B)** Differentially expressed genes from bulk RNA-seq comparing siDGk and siCONsiCON HepG2 cells, highlighting downregulation of *MAT1A*, a gene involved in methylation metabolism, and upregulation of interferon-stimulated genes *ISG15*, *ISG20*, and *OAS1* (DESeq2, adjusted *p* < 0.05). **(C)** Schematic of bisulfite-specific high-resolution melt analysis (MS-HRM) used to assess whether global methylation loss extends to CpG-rich promoter regions of interferon-stimulated genes. Genomic DNA was bisulfite-converted, amplified using bisulfite-specific primers targeting *ISG15* and *ISG20* promoter CpG regions, and analyzed by melt-curve profiling. Lower melting temperature and/or broader melt profiles reflect reduced or mixed methylation states. **(D)** MS-HRM analysis of *ISG15* and *ISG20* promoter methylation at 72 h posttransfection in siCON-, siDGk-, and siDNMT1-transfected HepG2 cells. Box plots show melt peak height (−d*F*/d*T*) per well. Statistical comparisons were performed using the Kruskal–Wallis test with Dunn’s *post hoc* correction; *p*-values are shown above brackets (*n* = 15 wells per group). **(E)** RT-qPCR validation showing that dAdo exposure is sufficient to recapitulate transcriptional changes observed following DGUOK depletion, including reduced MAT1A expression and increased *ISG15*, *ISG20*, and *OAS1* expression. HepG2 cells were treated with 1,000 µM dAdo or left unstimulated. Expression was normalized to ACTB and shown as fold-change relative to unstimulated controls. Statistical analysis was performed using unpaired two-tailed *t*-tests: MAT1A *p* = 0.0057, ISG15 *p* = 0.0205, ISG20 *p* = 0.0199, and OAS1 *p* = 0.0127 (*n* = 3 per group; mean ± SEM). **(F)** Flow cytometric analysis of BODIPY fluorescence intensity showing increased neutral lipid accumulation in siDGk-transfected cells compared with siCON, and following increasing concentrations of dAdo treatment. siDGk vs. siCON was analyzed using the Mann–Whitney *U* test (*U* = 4, *p* = 0.0260, *n* = 6 per group). dAdo dose-response data were analyzed using one-way ANOVA with Dunnett’s multiple comparisons test compared with 0 µM dAdo (F(3,8) = 16.87, *p* = 0.0008, *R*^2^ = 0.8635; 500 µM vs. 0 µM *p* = 0.0174, 1,000 µM vs. 0 µM *p* = 0.0004; *n* = 3 per group). Error bars represent mean ± SEM unless otherwise indicated. *p < 0.05, **p < 0.01, ***p < 0.001.

Metabolic disorders involving alterations in purines and purine metabolism, including deficiency of Adenosine Deaminase 1 (ADA1) Adenosine Deaminase 2 (ADA2) Sterile Alpha Motif domain and Histidine-Aspartate domain-containing protein 1 (SAMHD1) and others, are linked to spontaneous activation of innate immune pathways (18, 48–50). Purine metabolism intersects with the cellular methionine cycle and methyl-donor pathways, where altered purine balance can influence SAM-dependent trans-methylation reactions. Similarly, disruption of enzymes involved in methyl-donor metabolism, including SAM, AHCY, and GNMT pathway components, alters hepatic lipid metabolism (51, 52). To explore whether an imbalance in metabolism is sufficient to evoke immune and lipid-associated cellular responses in DGUOK-deficient hepatocytes, HepG2 cells were treated with dAdo, a bioactive purine nucleoside substrate of DGUOK. Global DNA methylation in siDGk and purine-treated cells, quantified by 5-mC ELISA, indicated that siDGk and dAdo-treated cells showed ~ 40% reduction in 5-mC content (*p* = 0.0264 and *p* = 0.0051, respectively; **Figure 5A**), consistent with diminished methyl-donor availability. These findings establish reduced DNA methylation as a shared feature of both DGUOK depletion and dAdo-mediated purine imbalance. RNA-seq analysis further showed decreased expression of *MAT1A*, a methyl-donor metabolism-associated gene, alongside increased expression of interferon-stimulated genes including *ISG15*, *ISG20*, and *OAS1* (**Figure 5B**). To determine whether global hypomethylation extends to ISG promoters, we performed bisulfite-based methylation-sensitive high-resolution melting (MS-HRM) analysis of CpG-rich regions within the *ISG15* and *ISG20* promoters (**Figure 5C**). At 72 h posttransfection, siDGk cells exhibited a significant decrease in methylation-associated MS-HRM signal at the *ISG15* promoter relative to siCON cells (*p* = 0.0175), accompanied by broadening of the melt curve toward lower temperatures, consistent with an increased proportion of hypomethylated amplicons (**Figure 5D**; **Supplementary Figure 13**). A similar trend was observed at the *ISG20* promoter (**Figure 5D**; **Supplementary Figure 13**). siDNMT1, included as a positive control for methylation loss, induced a significant reduction in methylation-associated signal within *ISG20* (*p* = 0.0024). These findings suggest that DGUOK depletion is associated with promoter hypomethylation at ISGs.

We asked whether dAdo-mediated purine stress is sufficient to reproduce the transcriptional changes observed with DGUOK depletion. dAdo treatment reduced *MAT1A* expression and increased *ISG15*, *ISG20*, and *OAS1* expression compared with unstimulated controls (*MAT1A*, *p* = 0.0057; *ISG15*, *p* = 0.0205; *ISG20*, *p* = 0.0199; *OAS1*, *p* = 0.0127; **Figure 5E**), recapitulating the transcriptional pattern observed in siDGk cells. Flow cytometric analysis further showed a dose-dependent increase in BODIPY fluorescence following dAdo treatment, paralleling the increase observed in siDGk cells (**Figure 5F**). To determine whether dAdo-induced lipid accumulation required IFN/innate immune signaling, HepG2 cells were transfected with siRNAs targeting IFNAR1, STING/TMEM173, or p65/RELA prior to dAdo treatment. dAdo significantly increased lipid droplet accumulation in siCON-transfected cells, but knockdown of IFNAR1, STING/TMEM173, or p65/RELA did not significantly reduce dAdo-induced lipid accumulation (**Supplementary Figure 14**). These data suggest that dAdo-induced lipid accumulation does not require these IFN/innate immune signaling nodes under the conditions tested.

Collectively, data show that purine dysregulation is sufficient to produce the immunometabolic phenotype triggered by DGUOK deficiency, supporting a direct functional link between purine imbalance, methyl-donor limitation, innate immune activation, and lipid accumulation.

## 4 Discussion

Our study identifies DGUOK as a previously unrecognized regulator of hepatocellular immunometabolism whose influence extends beyond its canonical role in maintaining mitochondrial nucleotide pools and mtDNA synthesis. We show that acute and early loss of DGUOK initiates a cell-intrinsic program characterized by robust type I interferon and ISG activation, derepression of HERV elements, and marked lipid droplet accumulation, all occurring while mtDNA copy number, respiratory chain protein expression, and mitochondrial ultrastructure remain intact. These early responses are mirrored by exogenous deoxyadenosine exposure, demonstrating that perturbation of purine balance alone is sufficient to elicit the combined immune and steatotic phenotypes observed in DGUOK-deficient cells. In contrast, chronic DGUOK loss produces the expected mtDNA depletion, respiratory chain insufficiency, and mitochondrial morphological abnormalities. Together, these findings support a two-stage model in which purine-driven epigenetic and immunometabolic reprogramming precedes, and may predispose hepatocytes to, subsequent mitochondrial failure.

Transcriptomic analysis provides insight into the molecular events underlying this early immunometabolic state. Acute DGUOK depletion triggered coordinated remodeling of purine, methionine/SAM, and methylation-associated pathways alongside broad induction of HERVs and ISGs. This pattern is consistent with a model in which impaired purine salvage disrupts the methionine cycle, reduces SAM availability, and diminishes DNA methylation capacity, thereby derepressing methylation-sensitive HERV loci. This mechanism is unlikely to require direct inhibition of DNMT catalytic activity by dAdo. Rather, prior work supports a model in which excess dAdo or altered purine metabolism perturbs intracellular methylation potential by altering the SAM/SAH balance, thereby limiting SAM-dependent methyltransferase reactions. In this context, the reduction in *MAT1A* expression observed in both siDGk- and dAdo-treated cells provides additional support for impaired methyl-donor pathway capacity as a contributor to global 5-mC loss. Consistent with this model, locus-specific MS-HRM analysis showed that DGUOK knockdown was associated with reduced methylation-associated signal at the *ISG15* promoter and a directionally similar trend at the *ISG20* promoter, supporting the idea that global methylation loss can extend to regulatory regions of selected ISGs. Although these data do not establish promoter hypomethylation as the sole driver of ISG induction, they provide targeted evidence that DGUOK depletion is linked to methylation changes at ISG-associated loci, potentially facilitating transcriptional derepression in the setting of purine and methyl-donor imbalance. The resulting double-stranded RNA-like retroelement transcripts can engage RIG-I-like receptors and TLR3, producing the type I interferon signature (22, 44, 53, 55, 56). The fact that deoxyadenosine supplementation alone induced DNA hypomethylation, ISG activation, and lipid accumulation strongly reinforces this mechanistic link and positions DGUOK as a metabolic checkpoint that integrates purine homeostasis with epigenetic and antiviral surveillance pathways.

A notable finding is the accumulation of neutral lipids in DGUOK-deficient hepatocytes prior to measurable impairment of mitochondrial mtDNA content, complex I protein expression, and mitochondrial structure. Although type I interferon signaling has been implicated in hepatic lipid remodeling, inhibiting IFN-β or silencing upstream signaling proteins failed to significantly reverse the lipid phenotype, suggesting that immune activation is not the primary driver. Similarly, silencing IFNAR1, STING/TMEM173, or p65/RELA did not significantly reduce dAdo-induced lipid accumulation, further supporting the interpretation that purine stress promotes lipid accumulation through mechanisms that are at least partly independent of canonical IFN/innate immune signaling. Instead, our data support a purine-SAM-lipid axis in which methyl-donor insufficiency favors hepatic steatosis. Indeed, purine-mediated reduction of the universal methyl-donor SAM availability, which has been described in diverse cellular systems (18, 57–59), provides a unifying explanation for immune and lipid phenotypes. In addition to epigenetic changes, reduced SAM constrains phosphatidylcholine synthesis (via the PEMT pathway) and VLDL export, favoring hepatic triglyceride storage (51, 60, 61). This mechanism aligns with clinical reports of steatosis in DGUOK-deficient patients who do not meet clinical criteria for mtDNA depletion, reinforcing the idea that altered one-carbon metabolism contributes directly to hepatic lipid dysregulation in this disease.

The innate immune activation observed here parallels the signatures associated with several other inborn errors of purine metabolism. Deficiencies in ADA1, ADA2, PNP, and SAMHD1 each produce spontaneous interferon responses through mechanisms involving purine imbalance, disrupted nucleotide catabolism, and aberrant nucleic acid sensing (18, 20, 48, 62–64). Our results extend this paradigm to a mitochondrial nucleoside-salvage enzyme and demonstrate that immunostimulatory consequences can arise upstream of overt mitochondrial dysfunction. These findings broaden the conceptual landscape of interferonopathies and suggest that purine dysregulation, regardless of cellular compartment, can serve as a unifying trigger for viral-mimicry-driven inflammatory programs.

The temporal separation between early immunometabolic remodeling and later mtDNA depletion provides a framework for understanding disease progression in DGUOK deficiency. Purine-driven epigenetic stress, chronic ISG signaling, and lipid accumulation could create a cellular environment that further predisposes hepatocytes to progressive mitochondrial instability. Once mtDNA copy number declines, cells may enter a second stage characterized by complex I deficiency, mitochondrial fragmentation, and bioenergetic failure, which are the hallmarks of advanced MDS. This layered model helps reconcile clinical observations in which inflammatory or steatotic changes precede overt mitochondrial dysfunction in affected infants.

These mechanistic insights carry important therapeutic implications. Current strategies for DGUOK deficiency focus primarily on restoring mtDNA content through deoxynucleotide supplementation or gene therapy (65, 66). Our findings suggest that targeting the upstream immunometabolic sequelae of purine disruption may provide additional benefit, particularly for patients who present after the neonatal period or with multisystem disease. Since JAKi effectively suppressed STAT1 phosphorylation but did not reverse lipid accumulation in DGUOK-depleted cells, canonical JAK–STAT signaling does not appear to be the primary driver of the steatotic phenotype. However, modulation of interferon/JAK–STAT signaling may still be useful for limiting inflammatory or antiviral immune consequences of DGUOK deficiency, particularly if chronic ISG activation contributes to tissue injury or interferes with therapeutic gene delivery (67–69). In contrast, strategies aimed at replenishing methyl donors, enhancing phosphatidylcholine synthesis, or normalizing purine turnover may be more directly relevant for correcting the metabolic and lipid-associated consequences of DGUOK disruption. Purine pathway normalization, potentially via restoring adenosine-deoxyadenosine catabolism or supporting salvage capacity, may correct the proximal metabolic trigger in hepatocytes and other affected cells (50, 70). These strategies could complement liver-directed gene therapy, particularly for patients who present beyond the neonatal window or with multisystem disease, where complete correction of mitochondrial dysfunction is challenging. Notably, the observed induction of an antiviral IFN-I program suggests that gene therapy for DGUOK deficiency may be limited by activation of innate immune antiviral programs that might interfere with current AAV-mediated gene delivery strategies (71–73).

Our conclusions are derived from human HepG2 hepatoma cells, which offer a controlled system for mechanistic dissection but do not fully recapitulate the complexity of primary hepatocytes or whole-organism physiology. Although the transcriptomic and methylation data strongly support impaired methyl-donor metabolism, direct quantification of SAM flux and base-resolution methylation profiling (6, 18, 54, 55, 57, 59, 74–76) will be important to confirm these pathways. Moreover, while early mitochondrial integrity was mostly preserved in our models, we cannot exclude subtle mtDNA instability or low-level mitochondrial nucleic acid release that may contribute to innate sensing through activation of the cGAS–STING pathway (77–80) and enhance HERV-mediated signals. Future studies should integrate metabolic methionine flux analyses, targeted inhibition of nucleic acid sensors, and *in vivo* models (81–84) to evaluate the physiological relevance of this early immunometabolic program and its contribution to disease progression.

In summary, we reveal that DGUOK loss initiates a purine-dependent, epigenetically mediated program that activates innate antiviral signaling and reconfigures lipid metabolism well before mitochondrial failure ensues. This purine-regulated immune–metabolic axis provides a unifying explanation for the inflammation and steatosis observed in DGUOK deficiency in the absence of clinical mtDNA reductions and reframes mitochondrial nucleoside salvage as a gatekeeper of hepatocellular homeostasis. By illuminating therapeutically targetable nodes upstream of mtDNA depletion, our findings broaden the mechanistic understanding of this fatal disorder and point to new avenues for intervention in mitochondrial liver disorders.

## Data Availability

The Bulk RNA Sequencing and HERV datasets generated for this study can be found at Gene Expression Omnibus under accession number GSE309813 (http://www.ncbi.nlm.nih.gov/geo/).
