# Supplementary material for: Deoxyguanosine kinase deficiency couples purine metabolism to innate immune activation and lipid accumulation in hepatocytes
Source: Front Immunol. 2026 Jul 15;17:1758569. doi: 10.3389/fimmu.2026.1758569 (PMC13416635; doi:10.3389/fimmu.2026.1758569)
Supplement: Supplementary file 1 [file DataSheet1.pdf]

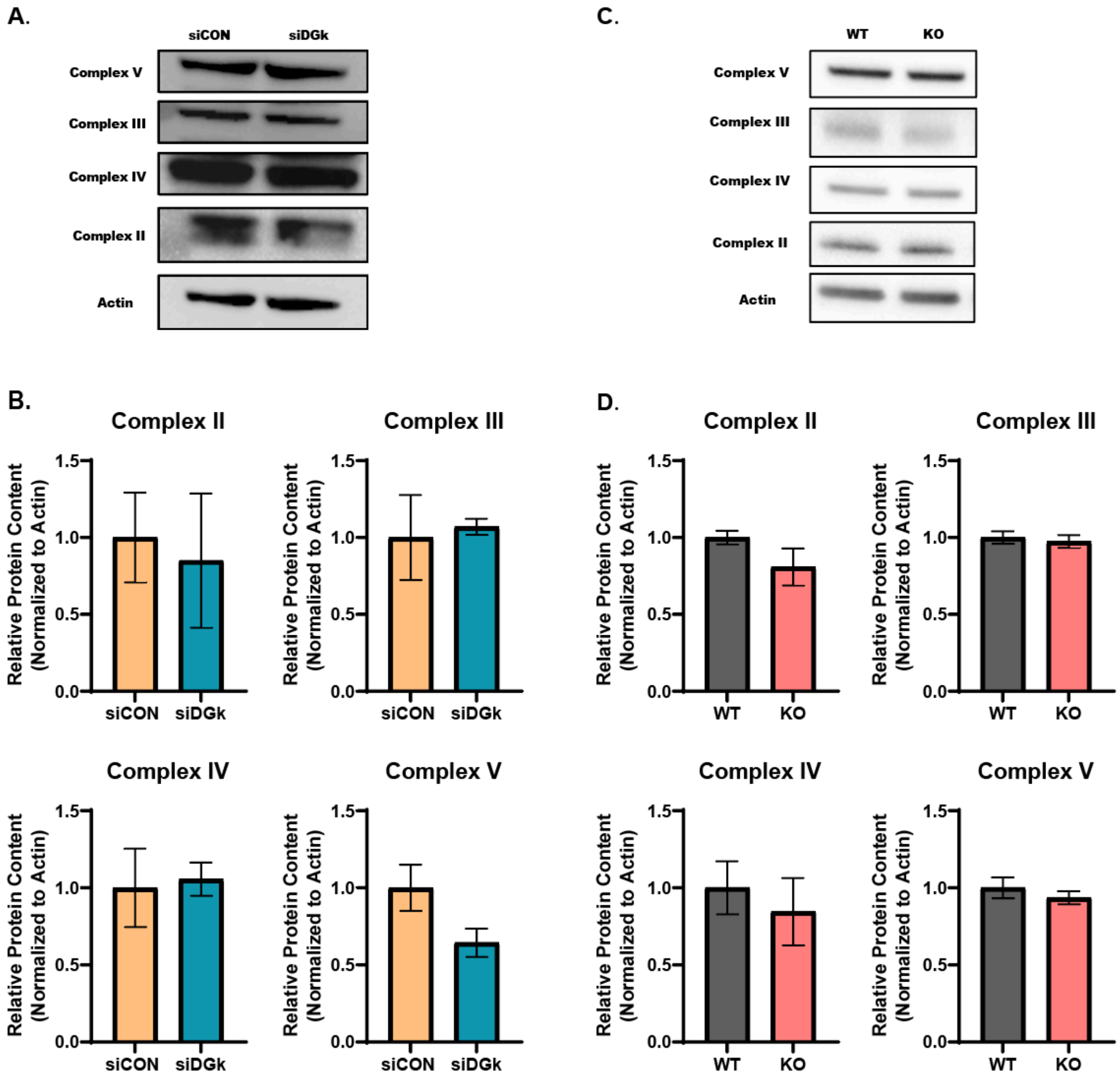

Figure S1. OXPHOS complex expression in transient and chronic DGUOK-deficient HepG2 cells. (A–B) Representative Western blots and quantification of mitochondrial oxidative phosphorylation (OXPHOS) complexes II–V in HepG2 cells transfected with siRNA targeting DGUOK (siDGk) or non-targeting control (siCON). Relative protein content was normalized to β-actin. Transient knockdown did not significantly alter the expression of Complex II (siCON = 1.00, siDGk = 0.9953, $p = 0.9900$), Complex III (siCON = 1.00, siDGk = 0.9442, $p = 0.297$), Complex IV (siCON = 1.00, siDGk = 0.8629, $p = 0.0787$), or Complex V (siCON = 1.00, siDGk = 0.8180, $p = 0.1932$). $n = 2$ biological replicates per group. (C–D) Representative Western blots and quantification of OXPHOS complexes II–V in DGUOK knockout (KO) HepG2 cells compared to wild-type (WT) controls. No significant changes were observed in Complex II (WT = 1.00, KO = 0.8082, $p = 0.209$), Complex III (WT = 1.00, KO = 0.9742, $p = 0.677$), Complex IV (WT = 1.00, KO = 0.8451, $p = 0.607$), or Complex V (WT = 1.00, KO = 0.9359, $p = 0.467$). $n = 3$ biological replicates per group. All data are presented as mean ± SEM and analyzed using unpaired two-tailed t-tests. No statistically significant differences were detected ($p > 0.05$).

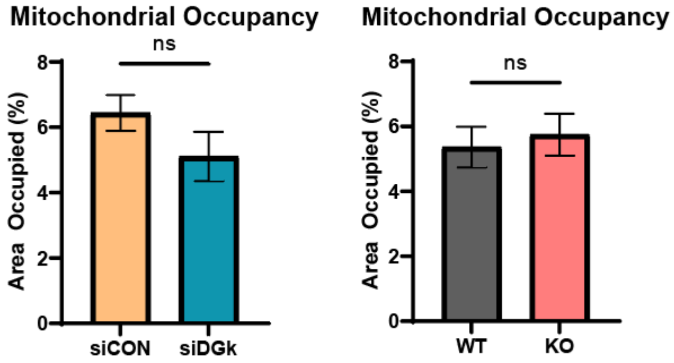

Figure S2. Transmission electron microscopy (TEM) analysis of mitochondrial occupancy in DGUOK-deficient hepatocytes. (A) Quantification of mitochondrial occupancy from TEM images of transient (siDGk) and chronic (KO) DGUOK-deficient HepG2 cells compared to controls. Mitochondrial occupancy was calculated as the percentage of cytoplasmic area occupied by mitochondria across 30 randomly selected cells per group. No significant differences were observed between siCON and siDGk cells (siCON = 6.439%, siDGk = 5.108%, $p = 0.1590$, $n = 30$ cells per group) or between WT and KO cells (WT = 5.364%, KO = 5.746%, $p = 0.6727$, $n = 30$ cells per group).

Data are presented as mean ± SEM and analyzed using unpaired two-tailed t-tests. ns, not significant ($p > 0.05$).

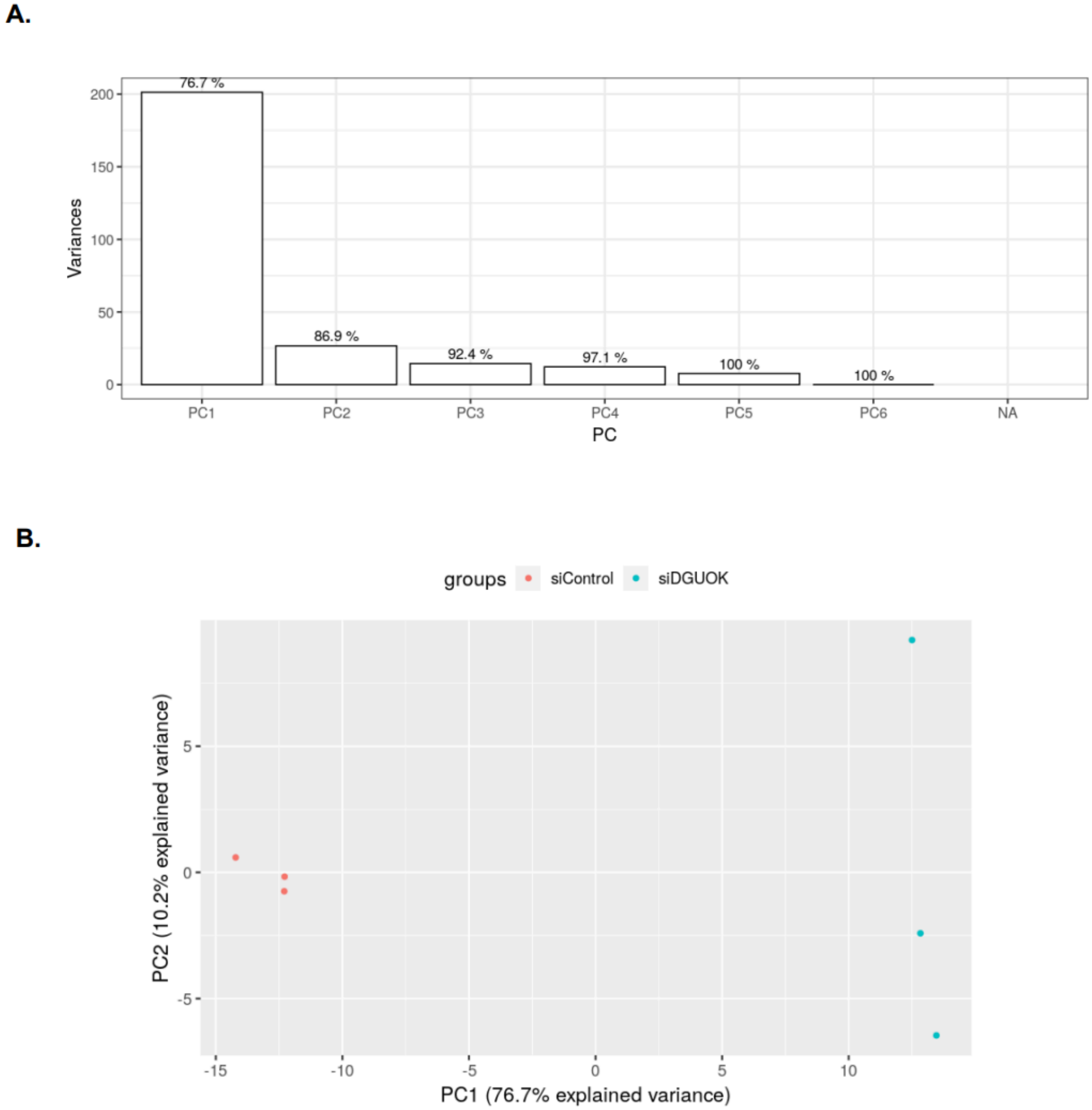

S3. Principal component analysis (PCA) of RNA-seq data (A) Scree plot showing the percentage of total variance explained by the rst six principal components. (B) PCA plot of PC1 versus PC2, which together explain 86.9% of the total variance. Samples from siControl and siDGk groups cluster separately, indicating distinct transcriptional proles between conditions.

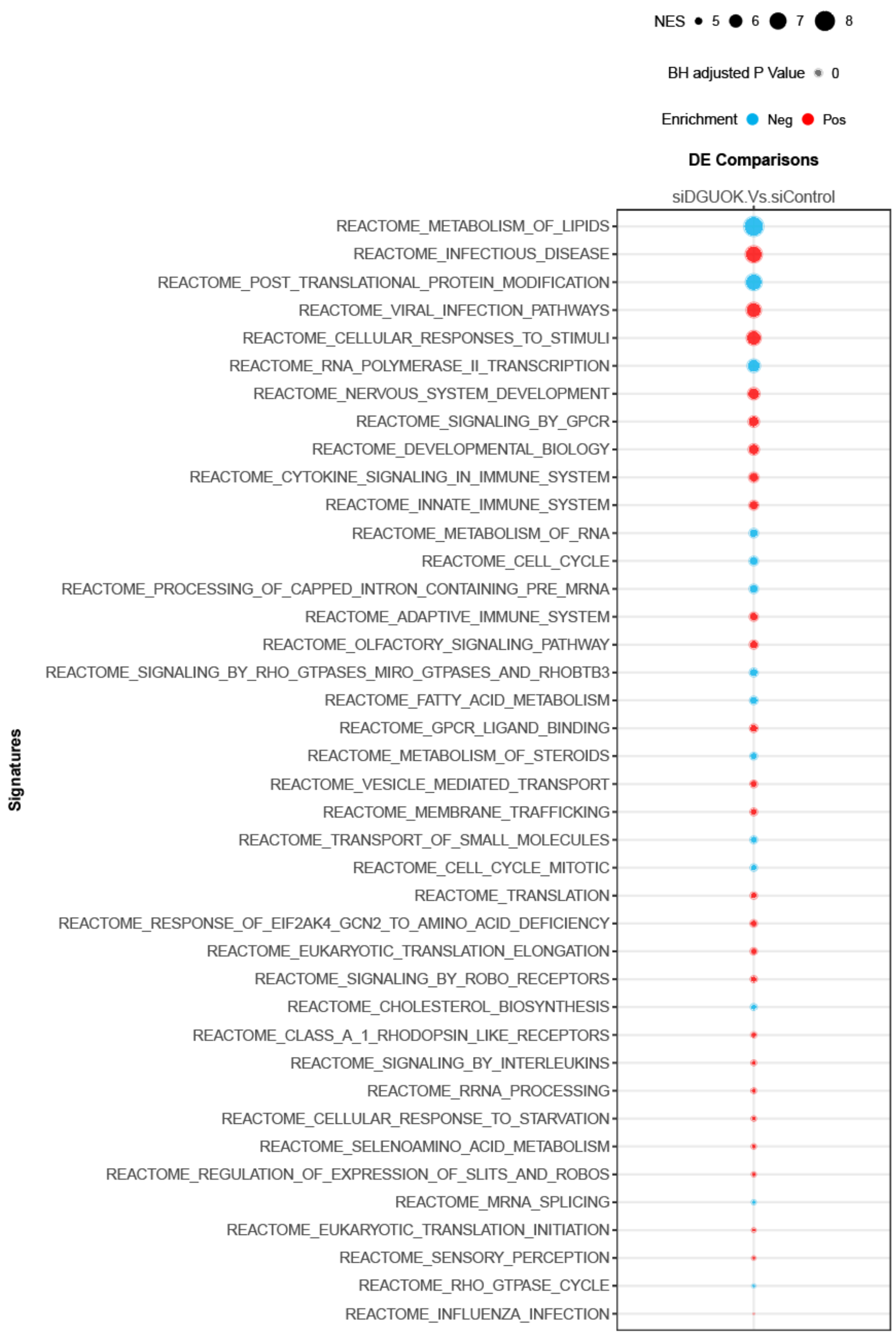

S4. Reactome Top Enriched Pathways in Gene Enrichment Set Analysis (GSEA). Dot plot summarizing the most significantly enriched Reactome pathways in siDGk versus siCON HepG2 cells (n = 3 per group). Upregulated immune and antiviral pathways (red) and downregulated lipid-metabolic pathways (blue) are shown with normalized enrichment scores (NES) proportional to dot size. Pathways include Interferon Signaling, Cytokine Signaling, Innate and Adaptive Immune System, Antiviral Mechanisms by IFN-Stimulated Genes, and Metabolism of Lipids, Fatty Acids, Steroids, and Cholesterol.

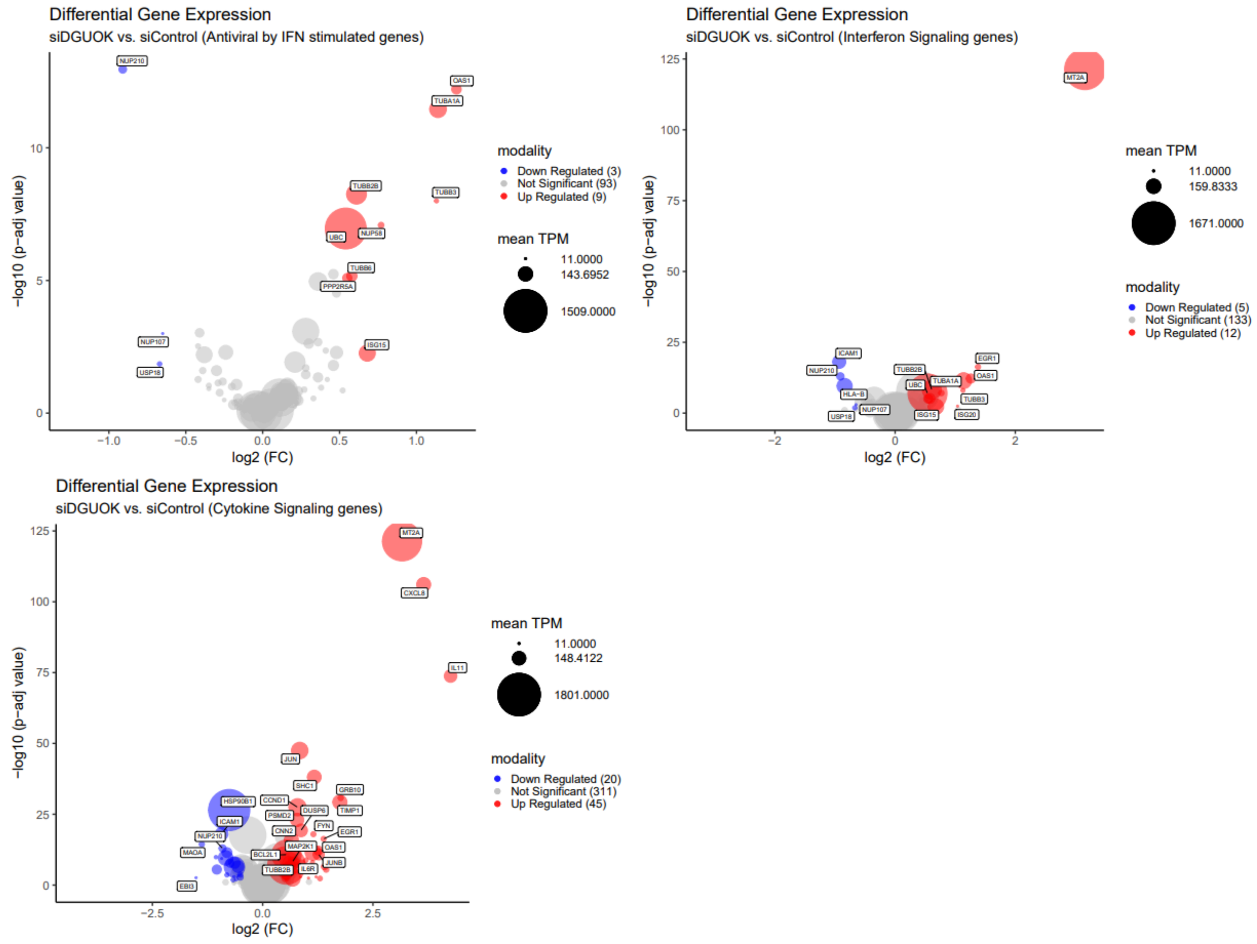

S5. Differential Gene Expression Volcano Plot of Immune-Related Enriched Pathways. Volcano plots showing transcriptional changes in antiviral (top), interferon signaling (middle), and cytokine signaling (bottom) gene sets in siDGk versus siCON HepG2 cells (n = 3 per group). Red and blue points represent significantly up- and downregulated genes, respectively (padj < 0.05, $|\log_2 FC| \geq 1.5$). Several interferon-stimulated genes (IFIT1, IFI44L, OAS1, RSAD2) are prominently upregulated, confirming activation of innate immune signaling following DGUOK knockdown.

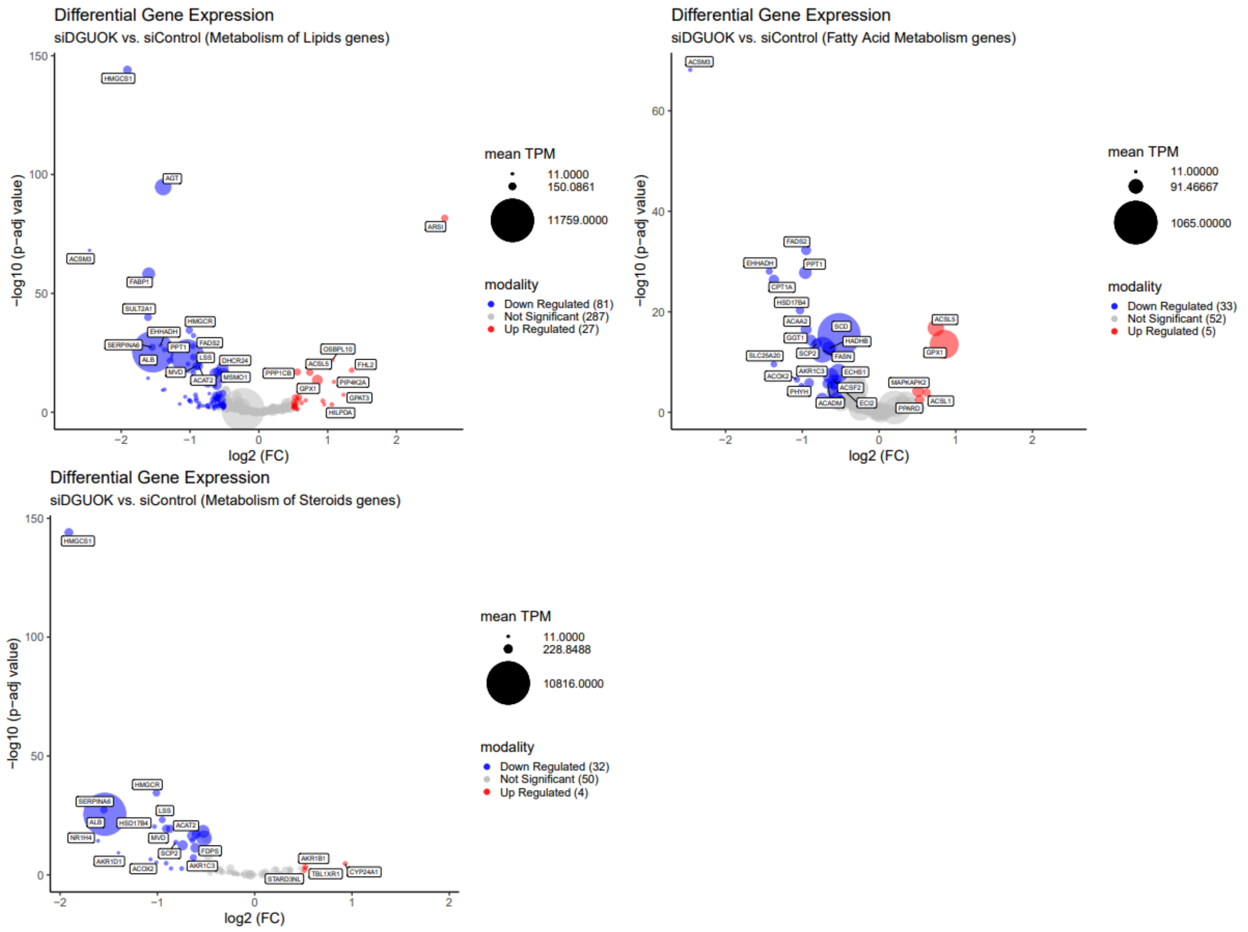

S6. **Lipid Related Plots.** Volcano plots depicting differential expression of genes in (top) Metabolism of Lipids, (middle) Fatty Acid Metabolism, and (bottom) Metabolism of Steroids pathways (n = 3 per group). Blue points indicate significant downregulation of key lipid-metabolic genes including HMGCR, ACACA, FASN, and SCD, consistent with suppressed lipid biosynthesis and β-oxidation programs in siDGk cells.

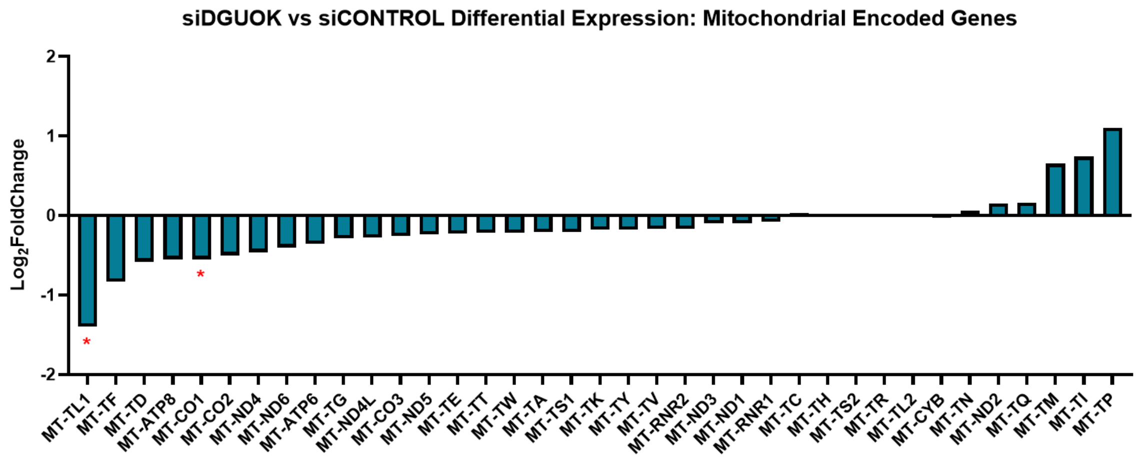

Figure S7. Mitochondrially encoded gene expression following transient DGUOK knockdown.

Bar plot showing differential expression ($\log_2$ fold change) of mitochondrially encoded genes in siDGk versus siCON HepG2 cells (n = 3 per group). Expression levels were obtained from bulk RNA-seq analysis. Among the 13 protein-coding mitochondrial genes, only protein encoding gene MT-CO1 was significantly downregulated ($\log_2$ FC = –0.56, padj = 0.0044), as well as tRNA gene MT-TL1. All other mitochondrial transcripts, including MT-ND, MT-CYB, and MT-ATP family members, remained unchanged, consistent with preserved mitochondrial mRNA stability at this early stage of DGUOK depletion.

Figure S8

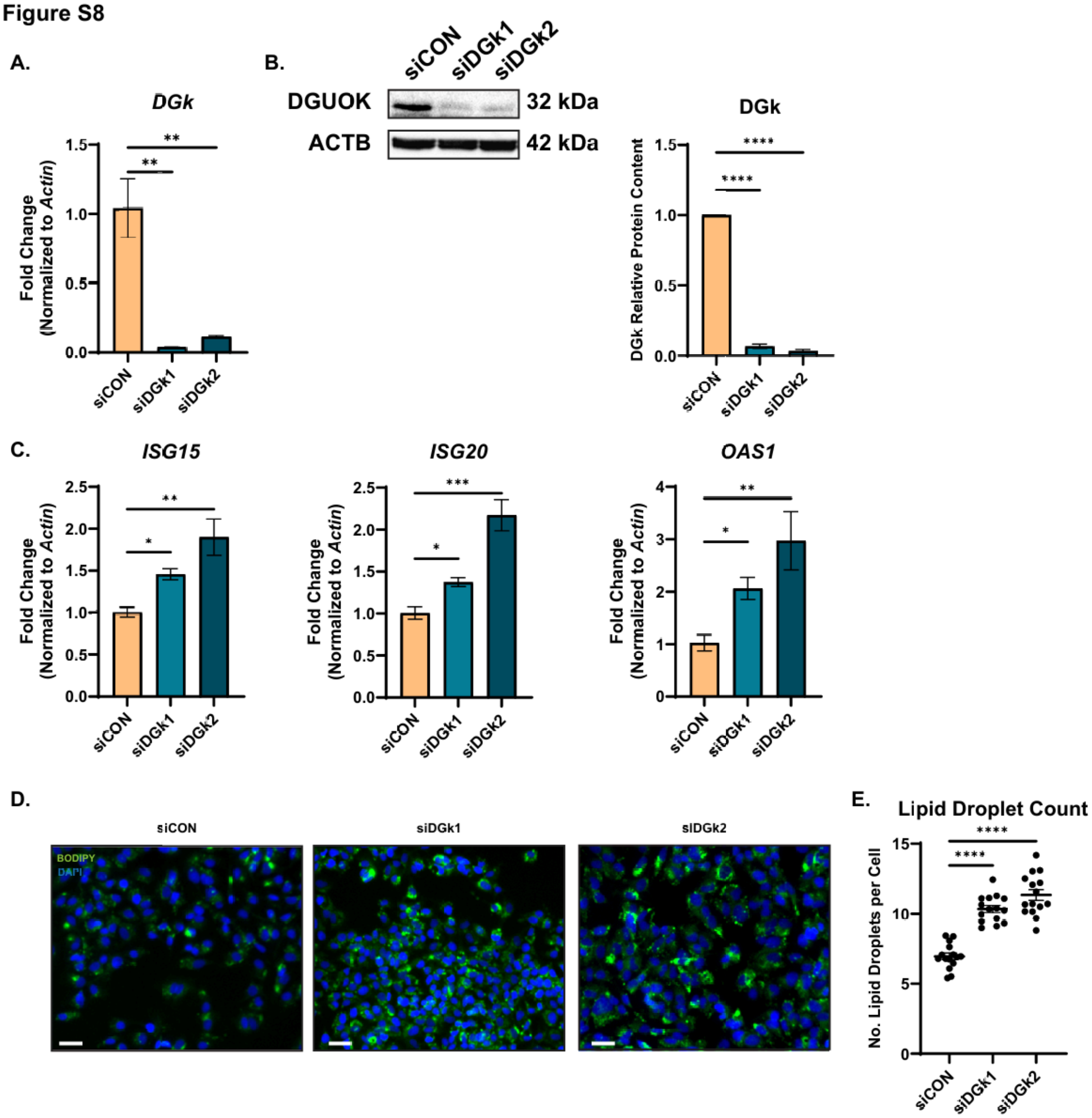
A.
DGk
Fold Change (Normalized to Actin)
siCON
siDGk1
siDGk2
B.
DGUOK
ACTB
32 kDa
42 kDa
DGk
DGk Relative Protein Content
C.
ISG15
ISG20
OAS1
D.
siCON
siDGk1
siDGk2
BODIPY
DAPI
E.
Lipid Droplet Count
No. Lipid Droplets per Cell

Figure S8. Independent siDGUOK siRNAs reproduce DGUOK knockdown, interferon-stimulated gene induction, and lipid accumulation.

(A) DGUOK mRNA expression was quantified by RT-qPCR in HepG2 cells transfected with two independent DGUOK-targeting siRNAs, siDGk1 and siDGk2, compared with siCON. Expression was normalized to ACTB and shown as fold change relative to siCON. Both siDGk1 and siDGk2 significantly reduced DGUOK mRNA expression ($n = 3$ per group; mean ± SEM; one-way ANOVA, $p = 0.0021$; Dunnett's multiple comparisons test versus siCON). **(B)** DGUOK protein levels were assessed by Western blot 72 h post-transfection in siCON-, siDGk1-, and siDGk2-transfected HepG2 cells. ACTB was used as a loading control. Quantification shows significant reduction of DGUOK protein levels by both independent siRNAs compared with siCON ($n = 2$ per group; mean ± SEM; one-way ANOVA, $p < 0.0001$; Dunnett's multiple comparisons test versus siCON). **(C)** RT-qPCR analysis of interferon-stimulated genes ISG15, ISG20, and OAS1 in siCON-, siDGk1-, and siDGk2-transfected HepG2 cells. Expression was normalized to ACTB and shown as fold change relative to siCON. Both independent DGUOK-targeting siRNAs increased ISG15, ISG20, and OAS1 expression compared with siCON (ISG15: one-way ANOVA, $p = 0.0047$; siDGk1 vs siCON $p = 0.0313$, siDGk2 vs siCON $p = 0.0030$; ISG20: one-way ANOVA, $p = 0.0008$; siDGk1 vs siCON $p = 0.0350$, siDGk2 vs siCON $p = 0.0005$; OAS1: one-way ANOVA, $p = 0.0093$; siDGk1 vs siCON $p = 0.0340$, siDGk2 vs siCON $p = 0.0063$; $n = 3$ per group; mean ± SEM; Dunnett's multiple comparisons test versus siCON). **(D)** Representative BODIPY 493/503 staining of neutral lipids (green) and DAPI nuclei (blue) in HepG2 cells transfected with siCON, siDGk1, or siDGk2. Scale bar = 100 µm. **(E)** Quantification of lipid droplets per cell from panel D, showing increased lipid droplet accumulation in cells transfected with either siDGk1 or siDGk2 compared with siCON ($n = 15$ fields/wells per group; mean ± SEM; one-way ANOVA, $p < 0.0001$; Dunnett's multiple comparisons test versus siCON). For all panels, *$P < 0.05$, **$P < 0.01$, ***$P < 0.001$, ****$P < 0.0001$.

Figure S9

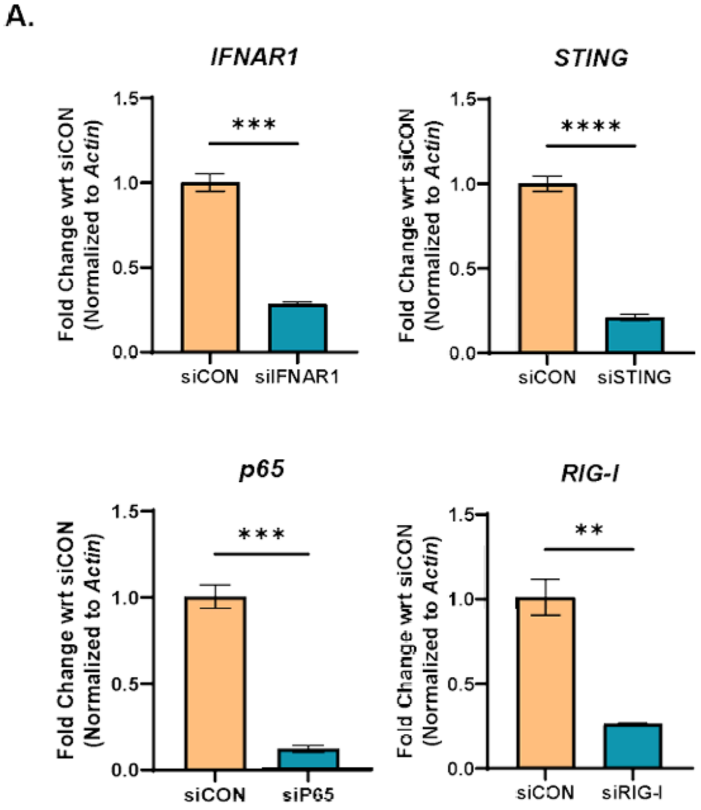
A.
IFNAR1
STING
p65
RIG-I
Fold Change wrt siCON (Normalized to Actin)
1.5
1.0
0.5
0.0
***
****
***
**
siCON
siIFNAR1
siSTING
siP65
siRIG-I

Figure S9. RT-qPCR validation of IFN-pathway siRNA knockdowns.

(A) RT-qPCR validation of siRNA-mediated knockdown of IFNAR1**,** STING/TMEM173**,** p65/RELA**,** and RIG-I/DDX58 in HepG2 cells. Cells were transfected with the indicated siRNAs, and target gene expression was quantified relative to siCON. Expression was normalized to ACTB and shown as fold change relative to siCON. All targeted siRNAs significantly reduced expression of their respective genes ($n = 3$ per group; mean ± SEM; unpaired two-tailed t-tests). $^{**}P < 0.01$, $^{***}P < 0.001$, $^{****}P < 0.0001$.

Figure S10

A.

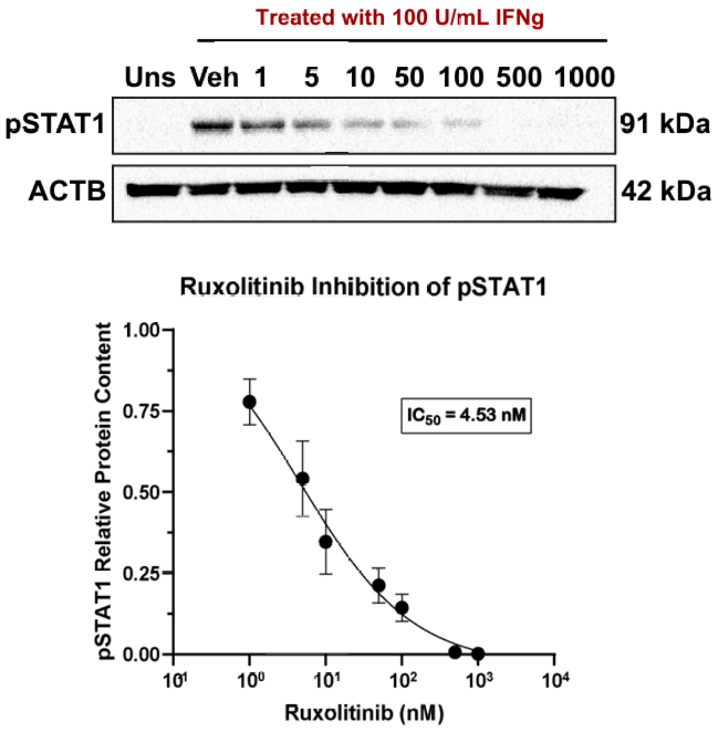

B.

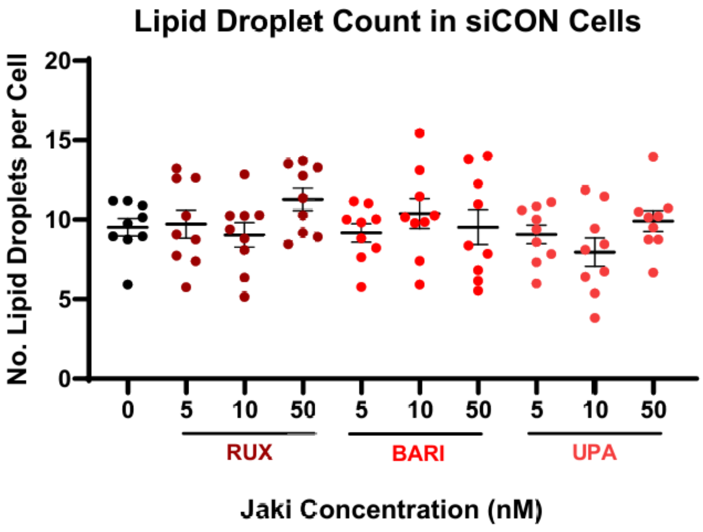

Figure S10. Ruxolitinib titration confirms pSTAT1 inhibition, and JAK inhibitors do not increase baseline lipid accumulation in HepG2 cells.

(A) HepG2 cells were stimulated with 100 U/mL IFNγ and treated with increasing concentrations of ruxolitinib (RUX; 1, 5, 10, 50, 100, 500, or 1000 nM) for 24 h. Phosphorylated STAT1 (pSTAT1) levels were assessed by Western blot, with ACTB used as a loading control. Quantification of pSTAT1 protein levels showed dose-dependent inhibition by ruxolitinib, with an estimated IC_{50} of 4.53 nM (n = 2; mean ± SEM). **(B)** Lipid droplet quantification in siCON-transfected HepG2 cells treated with vehicle or increasing concentrations of JAK inhibitors ruxolitinib (RUX), baricitinib (BARI), or upadacitinib (UPA). BODIPY 493/503 staining was used to quantify lipid droplets per cell, showing that JAK inhibitor treatment did not increase baseline lipid accumulation in control cells (n = 10 fields per condition; mean ± SEM; one-way ANOVA)

Figure S11

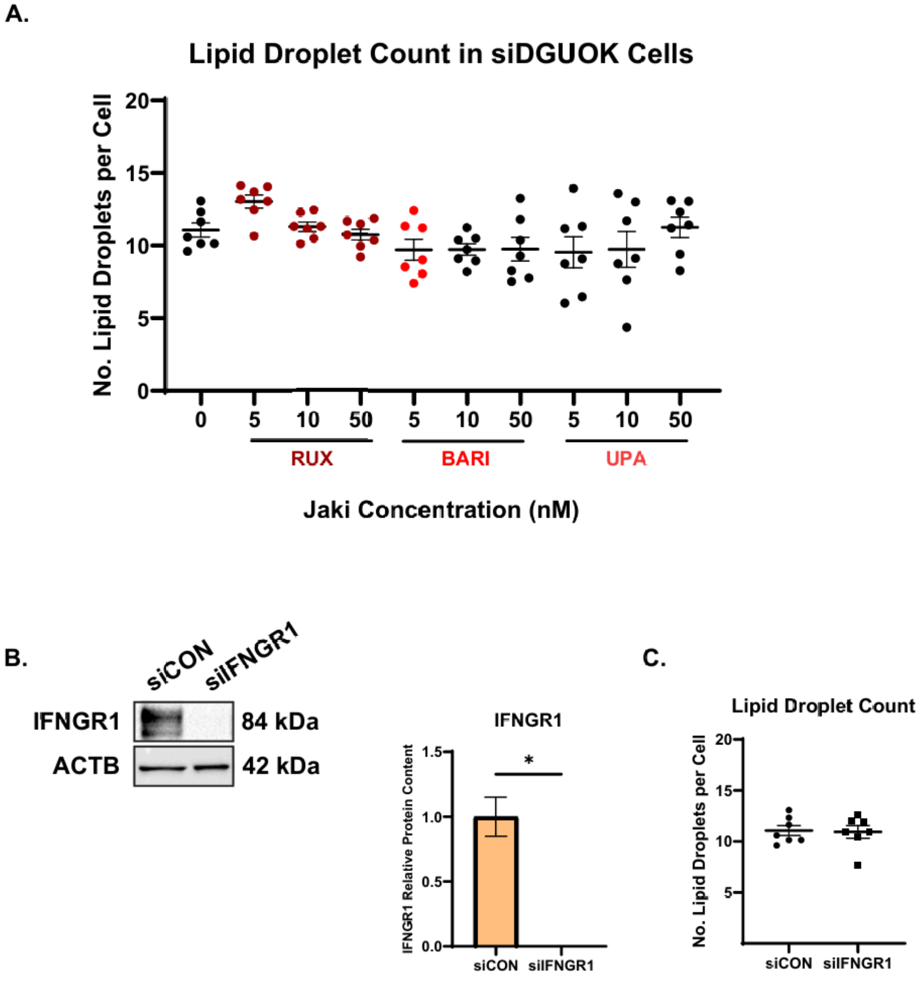

Figure S11. JAK inhibitor treatment and IFNGR1 silencing do not significantly reduce lipid accumulation in siDGUOK cells.

(A) Lipid droplet quantification in siDGUOK (siDGk)-transfected HepG2 cells treated with vehicle or increasing concentrations of JAK inhibitors ruxolitinib (RUX), baricitinib (BARI), or upadacitinib (UPA) at 5, 10, or 50 nM. BODIPY 493/503 staining was used to quantify lipid droplets per cell. JAK inhibitor treatment did not significantly reduce lipid droplet accumulation in siDGk cells (n = 7 fields per condition; mean ± SEM; one-way ANOVA). **(B)** IFNGR1 knockdown validation. IFNGR1 protein levels were assessed by Western blot in HepG2 cells transfected with siCON or siIFNGR1, with ACTB used as a loading control. Quantification shows reduced IFNGR1 protein levels following siIFNGR1 transfection (n = 2; mean ± SEM; unpaired two-tailed t-test). **(C)** BODIPY lipid droplet quantification following IFNGR1 silencing. Lipid droplet quantification showed that IFNGR1 silencing did not significantly alter lipid accumulation in siDGk-transfected HepG2 cells (n = 7 fields per condition; mean ± SEM; unpaired two-tailed t-test).*$P < 0.05$

Figure S12

A.

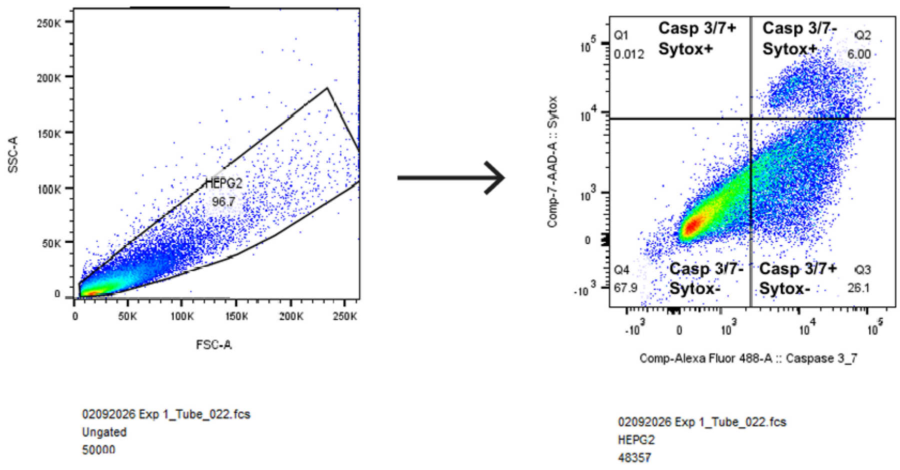

B.

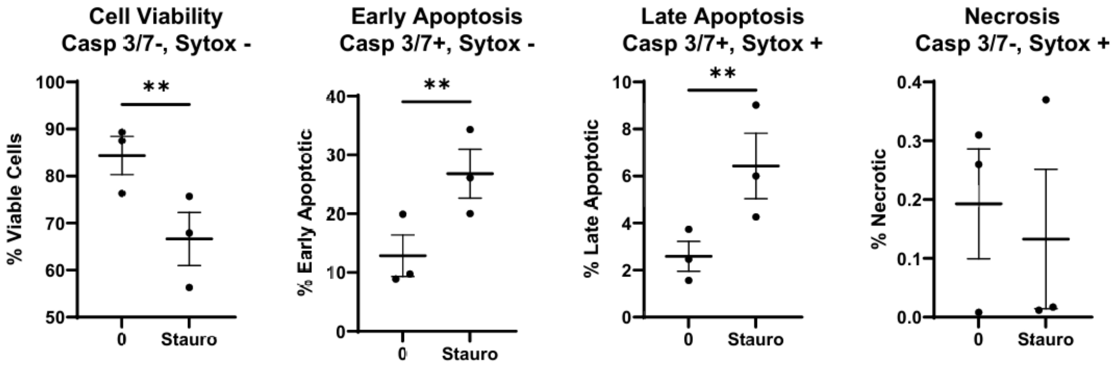

C.

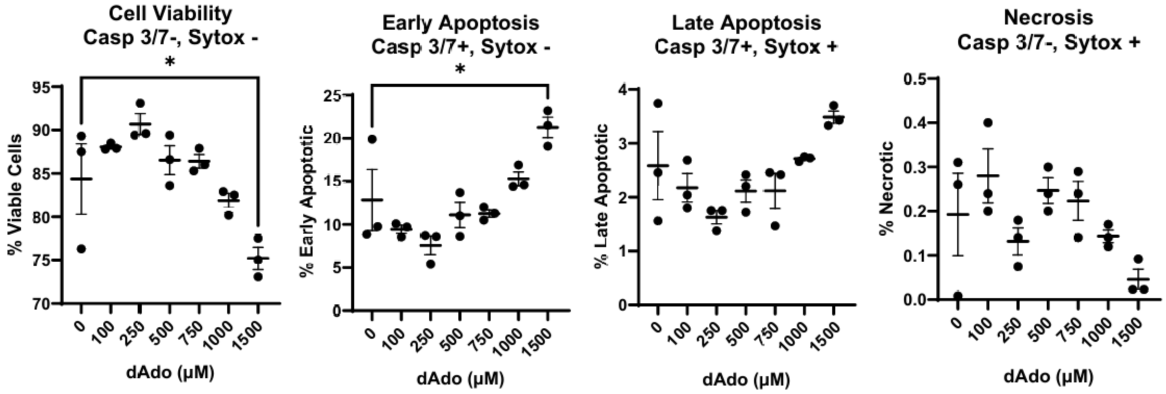

Figure S12. Flow cytometry-based validation of dAdo dose range using viability and apoptosis markers in HepG2 cells.

(A) Representative flow cytometry gating strategy for assessing cell viability and apoptosis using Caspase 3/7 and SYTOX staining. HepG2 cells were first gated based on forward and side scatter, followed by classification into viable cells (Caspase 3/7^{-} SYTOX^{-}), early apoptotic cells (Caspase 3/7^{+} SYTOX^{-}), late apoptotic cells (Caspase 3/7^{+} SYTOX^{+}), and necrotic cells (Caspase 3/7^{-} SYTOX^{+}). **(B)** Staurosporine-treated HepG2 cells were used as a positive control for apoptosis assay validation. Staurosporine treatment (2 µM, 24 h) significantly reduced the percentage of viable cells and increased early and late apoptotic populations compared with untreated controls, while necrosis was not significantly changed ($n = 3$ per condition; mean ± SEM; unpaired two-tailed t-tests). **(C)** Quantification of cell viability, early apoptosis, late apoptosis, and necrosis in HepG2 cells treated with increasing concentrations of deoxyadenosine (dAdo; 0, 100, 250, 500, 750, 1000, and 1500 µM). Viability was measured as the percentage of Caspase 3/7^{-} SYTOX^{-} cells, while early apoptosis, late apoptosis, and necrosis were measured as the percentage of cells in each corresponding gate. Statistical analysis was performed using one-way ANOVA with Dunnett's multiple comparisons test versus 0 µM dAdo ($n = 3$ per condition; mean ± SEM). Viability: 1500 µM vs 0 µM, $p = 0.0160$. Early apoptosis: 1500 µM vs 0 µM, $p = 0.0116$. $^{*}P < 0.05$, $^{**}P < 0.01$; ns, not significant.

Figure S13

A.

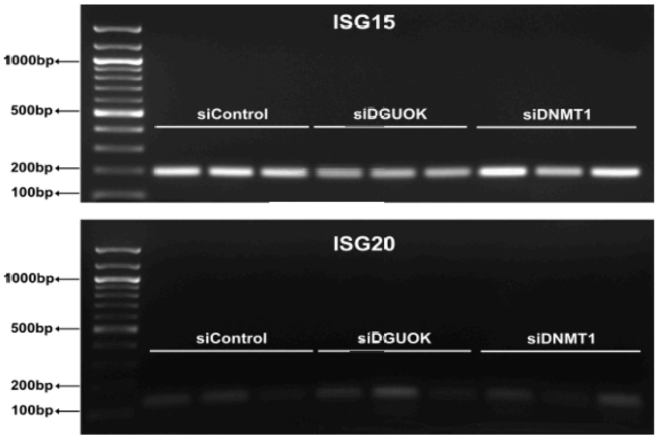

B.

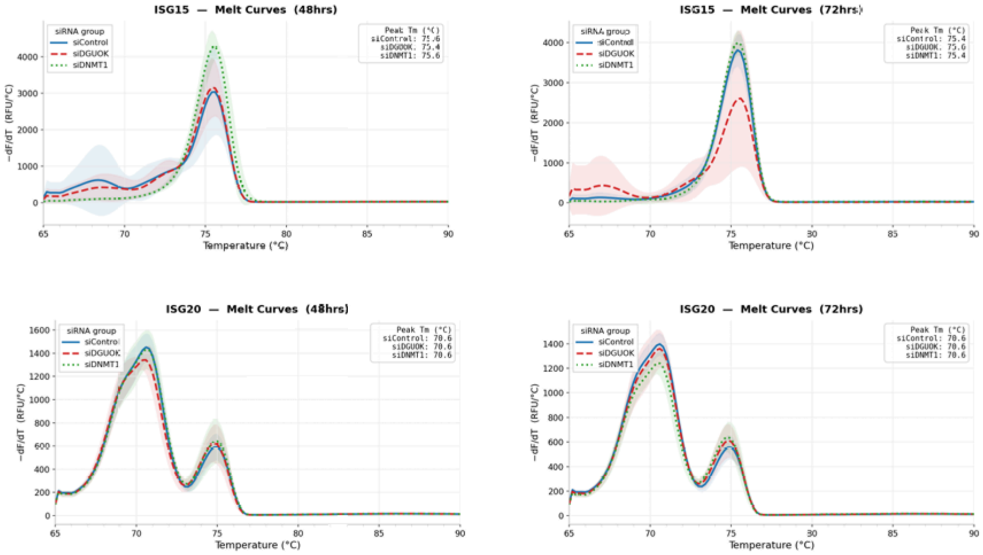

C.

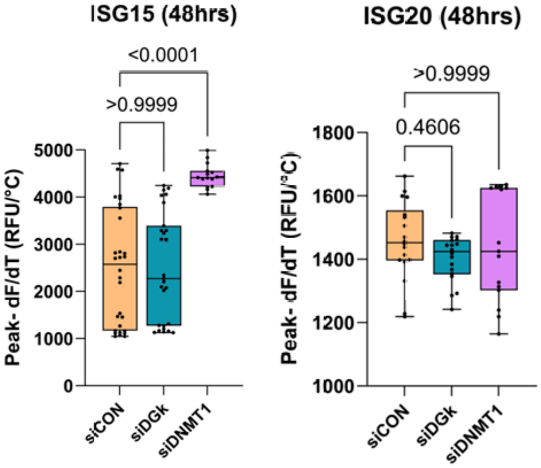

Figure S13. MS-HRM assay validation and 48 h promoter methylation analysis of ISG15 and ISG20.

(A) Bisulfite-specific PCR amplification of ISG15 and ISG20 promoter regions prior to MS-HRM analysis. Agarose gel electrophoresis of PCR products from HepG2 cells transfected with siCON, siDGUOK (siDGk), or siDNMT1 is shown, with three representative samples per group. ISG15 primers yielded a single specific band of approximately 200 bp across treatment groups, while ISG20 primers yielded a single specific band of approximately 150 bp. DNA ladder size markers are shown in the leftmost lane. Successful amplification of single-band products of the expected size confirmed primer specificity prior to downstream HRM analysis. **(B)** Representative derivative melt curves (-dF/dT) for ISG15 and ISG20 promoter amplicons at 48 h and 72 h post-transfection in siCON-, siDGk-, and siDNMT1-transfected HepG2 cells. Curves are shown as mean ± SD. **(C)** Quantification of MS-HRM melt peak height (-dF/dT) for ISG15 and ISG20 promoter amplicons at 48 h post-transfection. Box plots show peak -dF/dT amplitude per well. Statistical comparisons were performed using Kruskal-Wallis test with Dunn’s multiple comparisons test; p-values are shown above brackets (n = 15 wells per group).

Figure S14

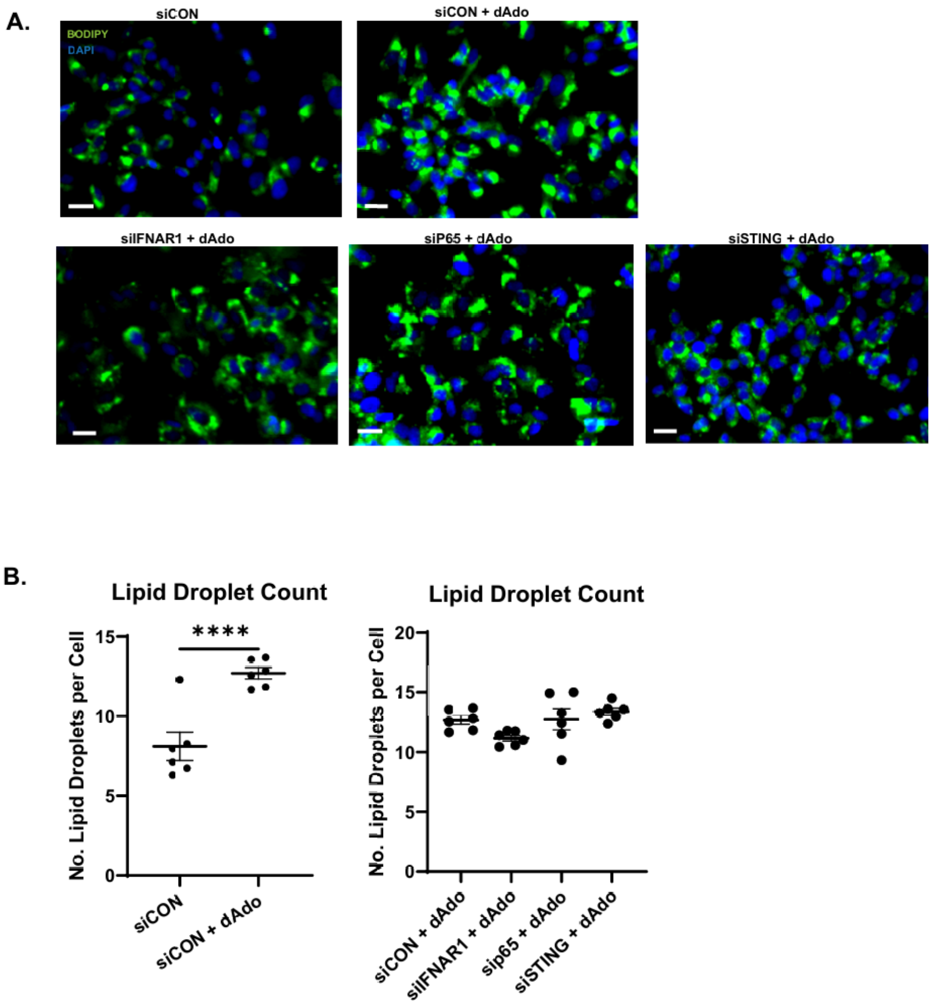

Figure S14. IFNAR1, p65, and STING silencing do not significantly reduce dAdo-induced lipid accumulation.

(A) Representative BODIPY 493/503 staining of neutral lipids (green) and DAPI nuclei (blue) in HepG2 cells treated with deoxyadenosine (dAdo) following siRNA-mediated silencing of IFNAR1, p65/RELA, or STING/TMEM173. Scale bar = 100 µm. **(B)** Quantification of lipid droplets per cell. dAdo treatment significantly increased lipid droplet accumulation in siCON-transfected cells compared with untreated siCON controls (n = 6 fields per condition; mean ± SEM; unpaired two-tailed t-test). In dAdo-treated cells, silencing of IFNAR1, p65/RELA, or STING/TMEM173 did not significantly reduce lipid droplet accumulation compared with siCON + dAdo (n = 6 fields per condition; mean ± SEM; one-way ANOVA). ****$P < 0.0001$; ns, not significant.
