# Supplementary material for: Deoxyguanosine kinase deficiency couples purine metabolism to innate immune activation and lipid accumulation in hepatocytes
Source: Front Immunol. 2026 Jul 15;17:1758569. doi: 10.3389/fimmu.2026.1758569 (PMC13416635; doi:10.3389/fimmu.2026.1758569)
Supplement: Supplementary file 2 [file DataSheet2.pdf]

Gene symbol	TaqMan probe ID
DGUOK	Hs00361549_m1
ACTB	Hs01060665_g1
HBB	Hs00758889_s1
MT-CO1	Hs02596864_g1
MT-ND1	Hs02596873_s1
ISG15	Hs01921425_s1
ISG20	Hs00158122_m1
MAT1A	Hs01547962_m1
OAS1	Hs00973635_m1
IFNB1	Hs01077958_s1

Supplementary Table 1. TaqMan Probes

This table lists all TaqMan Gene Expression Assays used for quantitative RT-PCR validation of key transcripts identified by RNA-seq in HEPG2 cells.

Gene Name	Complex	Log_2FC	padj	Clinical Depletion in DGUOK Patients
MT-ND1	I	–0.10	0.85	↓ mtDNA < 20%
MT-ND2	I	+0.15	0.71	↓ mtDNA < 20%
MT-ND3	I	–0.10	0.53	↓ mtDNA < 20%
MT-ND4	I	–0.46	0.16	↓ mtDNA < 20%
MT-ND4L	I	–0.28	0.41	↓ mtDNA < 20%
MT-ND5	I	–0.24	0.56	↓ mtDNA < 20%
MT-ND6	I	–0.40	0.36	↓ mtDNA < 20%
MT-CO1	IV	–0.56	0.0044*	↓ mtDNA < 20%
MT-CO2	IV	–0.50	0.062	↓ mtDNA < 20%
MT-CO3	IV	–0.26	0.27	↓ mtDNA < 20%
MT-CYB	III	+0.01	0.99	↓ mtDNA < 20%
MT-ATP6	V	–0.36	0.35	↓ mtDNA < 20%
MT-ATP8	V	–0.55	0.13	↓ mtDNA < 20%

Table 2. Summary of mitochondrially encoded gene expression changes in siDGk versus siCON HepG2 cells and clinical depletion patterns in DGUOK-deficient patients. Table summarizing log_2 fold changes and adjusted p-values for mitochondrially encoded oxidative phosphorylation genes (Complex I–V) from RNA-seq analysis. For clinical comparison, reported mtDNA depletion levels (< 20 %) in DGUOK-deficient patient liver tissue are included(PMID:39079226).

Gene Symbol	Rank in Gene List	Rank Metric Score	Running Enrichment Score	Core Enrichment	Protein Encoded
RCL1	147	13.9208	0.0006	Yes	RNA 3′-phosphate cyclase-like protein (rRNA processing factor)
RIOK3	498	5.8539	-0.0048	Yes	Ser/Thr protein kinase RIO3
UTP4	563	5.2924	-0.0017	Yes	UTP4/Cirhin, SSU processome component
RPL10	619	4.8539	0.0016	Yes	60S ribosomal protein L10
RPS11	684	4.4318	0.0047	Yes	40S ribosomal protein S11
RPL22L1	685	4.4318	0.0096	Yes	60S ribosomal protein L22-like 1
RPL31	883	3.6778	0.0088	Yes	60S ribosomal protein L31
RPL7A	893	3.6576	0.0135	Yes	60S ribosomal protein L7a
RPS25	917	3.5850	0.0178	Yes	40S ribosomal protein S25
RPS3A	940	3.5229	0.0221	Yes	40S ribosomal protein S3A

XRN2	956	3.4815	0.0266	Yes	5′→3′ exoribonuclease 2
RPL15	989	3.4202	0.0306	Yes	60S ribosomal protein L15
RPL9	1030	3.2518	0.0344	Yes	60S ribosomal protein L9
FCF1	1259	2.7212	0.0326	Yes	FCF1, SSU processome factor
RPL37	1392	2.4948	0.0337	Yes	60S ribosomal protein L37
RPL26	1471	2.3768	0.0363	Yes	60S ribosomal protein L26
TRMT10C	1621	2.1805	0.0369	Yes	tRNA methyltransferase 10C (MRPP1), mitochondrial
RPS15A	1645	2.1549	0.0412	Yes	40S ribosomal protein S15a
RPL8	1675	2.1249	0.0453	Yes	60S ribosomal protein L8
RPL39	1706	2.0969	0.0494	Yes	60S ribosomal protein L39

Supplementary Table 4-A. Reactome "rRNA Processing" — Core Enrichment (Top 20).

This table lists the top 20 leading-edge genes contributing to enrichment of the Reactome "rRNA Processing" pathway (R-HSA-72312) identified by GSEA preranked analysis comparing siDGUOK and siCON HEPG2 cells. Transient DGUOK knockdown triggered upregulation of nucleolar and ribosome-biogenesis genes including *RCL1*, *UTP4*, and multiple ribosomal protein subunits (*RPL10, RPS11, RPL26*), indicating increased RNA processing and translational turnover prior to mtDNA depletion. Positive rank-metric scores correspond to genes upregulated

in the siDGUOK condition, reflecting elevated ribosome biogenesis potentially linked to the observed interferon-stimulated transcriptional state. Functional enrichment within this category aligns with early adaptive remodeling of RNA metabolism secondary to nucleotide imbalance.

Gene Symbol	Rank in Gene List	Rank Metric Score	Running Enrichment Score	Core Enrichment	Protein Encoded
HMGA1	8	80.4559	0.0011	Yes	High mobility group AT-hook 1 (chromatin architectural factor)
PSMD2	57	25.0555	0.0009	Yes	26S proteasome regulatory subunit, non-ATPase 2
FYN	75	19.8539	0.0017	Yes	Src family tyrosine kinase Fyn
PPP1CB	82	18.7959	0.0029	Yes	Protein phosphatase 1 catalytic subunit beta (Ser/Thr phosphatase)
TAF7	104	17.0915	0.0036	Yes	TFIID subunit TAF7 (general transcription factor)
ITCH	126	14.6778	0.0042	Yes	E3 ubiquitin-protein ligase ITCH
TUBA1A	157	13.1024	0.0047	Yes	Tubulin alpha-1A
AP1S3	167	12.6576	0.0057	Yes	AP-1 adaptor complex subunit sigma-3
BCL2L1	175	12.3372	0.0068	Yes	Bcl-xL (BCL2-like 1), anti-apoptotic regulator
EGFR	192	11.4949	0.0076	Yes	Epidermal growth factor receptor (RTK)

TUBB	194	11.3665	0.0089	Yes	Tubulin beta chain
ATP1A1	198	11.3098	0.0102	Yes	Na^+/K^+-ATPase alpha-1 subunit
SH3KBP1	224	10.4559	0.0107	Yes	CIN85 adaptor (endocytosis/signaling)
IL6R	229	10.2676	0.0119	Yes	Interleukin-6 receptor alpha chain
SLC25A6	230	10.2596	0.0132	Yes	ADP/ATP translocase 3 (ANT3), mitochondrial carrier
TUBB2B	255	9.7212	0.0138	Yes	Tubulin beta-2B
TUBB3	269	9.4318	0.0148	Yes	Tubulin beta-3
SDC1	272	9.3279	0.0160	Yes	Syndecan-1, heparan sulfate proteoglycan
IRAK1	275	9.3010	0.0173	Yes	IL-1 receptor-associated kinase 1 (Ser/Thr kinase)
NUP58	311	8.4685	0.0175	Yes	Nucleoporin 58 (Nup58), nuclear pore complex

Supplementary Table 3-B. Reactome “Viral Infection Pathways” — Core Enrichment (Top 20).

This table summarizes the top 20 leading-edge genes from the Reactome viral infection–related pathways, highlighting strong induction of interferon-responsive and stress-signaling genes in siDGUOK cells. Genes such as *HMGA1*, *EGFR*, *IRAK1*, *IL6R*, and *ITCH* show positive rank-metric scores, consistent with activation of antiviral signaling, cytokine receptor pathways, and proteasome-mediated protein turnover. These transcriptional changes reflect an interferon-stimulated, antiviral-like cellular environment following loss of DGUOK, preceding mitochondrial dysfunction. The enrichment supports that purine disequilibrium activates innate immune programs resembling viral mimicry.

Gene Symbol	Rank in Gene List	Rank Metric Score	Running Enrichment Score	Core Enrichment	Protein Encoded

OSBPL6	28054	-0.4089	-0.3501	Yes	Oxysterol-binding protein-related protein 6 (ORP6)
AKR1C2	28268	-0.4318	-0.3499	Yes	Aldo-keto reductase family 1 member C2 (3α-HSD)
TGS1	28430	-0.4559	-0.3481	Yes	Trimethylguanosine synthase 1 (cap hypermethylase)
ACACB	28694	-0.4949	-0.3493	Yes	Acetyl-CoA carboxylase beta
PPARA	28899	-0.5229	-0.3488	Yes	PPAR-α nuclear receptor
SEC24B	29134	-0.5686	-0.3491	Yes	COPII coat subunit Sec24B
SEC23A	29206	-0.5850	-0.3447	Yes	COPII coat subunit Sec23A
PLPP6	29259	-0.5850	-0.3396	Yes	Phospholipid phosphatase 6
FDX1	29375	-0.6198	-0.3365	Yes	Ferredoxin-1 (mitochondrial electron transfer for steroidogenesis)
GC	29683	-0.6990	-0.3390	Yes	Vitamin D-binding protein (group-specific component)

LDLRAP1	29854	-0.7447	-0.3375	Yes	LDL receptor adaptor protein 1
CYP7B1	29869	-0.7447	-0.3313	Yes	Oxysterol 7-α-hydroxylase
SEC24A	30560	-1.0000	-0.3452	Yes	COPII coat subunit Sec24A
AKR1C4	30620	-1.0088	-0.3404	Yes	Aldo-keto reductase family 1 member C4 (3α-HSD)
TBL1X	30695	-1.0458	-0.3360	Yes	Transducin beta-like 1, X-linked (coregulator)
NFYC	30716	-1.0555	-0.3300	Yes	NF-Y transcription factor subunit gamma
CHD9	30776	-1.0809	-0.3252	Yes	Chromodomain helicase DNA-binding protein 9
STARD5	30786	-1.0862	-0.3189	Yes	StAR-related lipid transfer protein 5
CYP17A1	31108	-1.2676	-0.3218	Yes	17-α-hydroxylase/17,20-lyase
LBR	31178	-1.3188	-0.3173	Yes	Lamin B receptor (sterol-reductase–like; inner nuclear membrane)

Supplementary Table 4-C. Reactome "Metabolism of Steroids" — Core Enrichment (Top 20).

The "Metabolism of Steroids" pathway exhibited strong negative enrichment, with leading-edge genes including *AKR1C2*, *FDX1*, *CYP7B1*, and *CYP17A1* downregulated in siDGUOK cells.

These genes govern cholesterol, bile acid, and steroid hormone synthesis, processes typically dependent on adequate mitochondrial function and acetyl-CoA availability. Downregulation of these genes parallels the lipidomic and morphological evidence of impaired fatty acid oxidation and reduced steroidogenic activity described in the results. Together, these findings suggest that loss of DGUOK disrupts hepatocellular sterol homeostasis independently of mtDNA depletion.

Gene Symbol	Rank in Gene List	Rank Metric Score	Running Enrichment Score	Core Enrichment	Protein Encoded

ARNT	28262	-0.4318	-0.2743	Yes	Aryl hydrocarbon receptor nuclear translocator (HIF-1β)
AKR1C2	28268	-0.4318	-0.2731	Yes	Aldo-keto reductase 1C2 (3α-HSD)
LTC4S	28289	-0.4318	-0.2723	Yes	Leukotriene C4 synthase
PIK3C2G	28341	-0.4437	-0.2725	Yes	Class II PI3K, catalytic subunit C2-γ
GBA3	28382	-0.4437	-0.2723	Yes	Cytosolic β-glucosidase
PRKAG2	28411	-0.4559	-0.2718	Yes	AMPK γ2 regulatory subunit
MTMR4	28413	-0.4559	-0.2704	Yes	Myotubularin-related phosphatase 4
TGS1	28430	-0.4559	-0.2695	Yes	Trimethylguanosine synthase 1
ACSBG1	28520	-0.4685	-0.2708	Yes	Very-long-chain acyl-CoA synthetase (bubblegum 1)
PLA2G5	28529	-0.4685	-0.2697	Yes	Group V secreted phospholipase A2

CYP4B1	28541	-0.4685	-0.2686	Yes	Cytochrome P450 4B1 (ω-hydroxylase)
FUT1	28556	-0.4685	-0.2677	Yes	Fucosyltransferase 1 (H-antigen synthesis)
PIK3C2B	28623	-0.4815	-0.2683	Yes	Class II PI3K, catalytic subunit C2-β
PTEN	28646	-0.4815	-0.2675	Yes	PTEN lipid/protein phosphatase (PI3K/Akt brake)
RUFY1	28648	-0.4815	-0.2662	Yes	RUN and FYVE domain-containing protein 1 (endosomal trafficking)
TNFAIP8L1	28654	-0.4815	-0.2649	Yes	TNFAIP8-like 1 (TiPEL1; lipid-binding/apoptosis)
ACACB	28694	-0.4949	-0.2647	Yes	Acetyl-CoA carboxylase β
SMPD4	28709	-0.4949	-0.2638	Yes	Neutral sphingomyelinase 3/4 (sphingomyelin phosphodiesterase)
ACSL6	28719	-0.4949	-0.2627	Yes	Long-chain acyl-CoA synthetase 6
ACOT6	28736	-0.4949	-0.2618	Yes	Acyl-CoA thioesterase 6

Supplementary Table 4-D. Reactome “Metabolism of Lipids” — Core Enrichment (Top 20).

This table details the top 20 downregulated genes driving negative enrichment of the “Metabolism of Lipids” pathway. Core genes such as *PPARA*, *ACACB*, *PLA2G5*, *PTEN*, and *PRKAG2* exhibited negative rank-metric scores, consistent with suppression of fatty acid β-oxidation, phospholipid remodeling, and peroxisomal transport. The collective downregulation of lipid metabolic genes supports the accumulation of intracellular lipids observed by BODIPY and Oil Red O staining in siDGUOK HEPG2 cells and reinforces that metabolic reprogramming occurs prior to measurable mitochondrial genome loss.

Gene Symbol	Rank in Gene List	Rank Metric Score	Running Enrichment Score	Core Enrichment	Protein Encoded

MT2A	3	124.6383	0.0039	Yes	Metallothionein-2A (metal/oxidative-stress binder)
EGR1	91	18.1427	0.0054	Yes	Early growth response 1 transcription factor
OAS1	148	13.8861	0.0077	Yes	2′-5′-oligoadenylate synthetase 1 (RNase L pathway)
TUBA1A	157	13.1024	0.0115	Yes	α-Tubulin 1A (microtubules)
TUBB2B	255	9.7212	0.0126	Yes	β-Tubulin 2B
TUBB3	269	9.4318	0.0162	Yes	β-Tubulin 3
YBX1	285	8.9586	0.0198	Yes	Y-box binding protein 1 (mRNA/DNA binding)
NUP58	311	8.4685	0.0231	Yes	Nucleoporin 58 (nuclear pore)
UBC	322	8.3468	0.0268	Yes	Polyubiquitin C (ubiquitin precursor)
PLCG1	435	6.4685	0.0275	Yes	Phospholipase C-γ1 (signal transduction)

TUBB6	444	6.3979	0.0313	Yes	β-Tubulin 6
PPP2R5A	452	6.3188	0.0351	Yes	PP2A regulatory subunit B56-α
TUBA1C	469	6.1739	0.0386	Yes	α-Tubulin 1C
NUP43	519	5.6990	0.0412	Yes	Nucleoporin 43
HSPA2	707	4.3098	0.0396	Yes	HSP70 family chaperone (HSPA2)
TUBA1B	772	4.0915	0.0417	Yes	α-Tubulin 1B
UBE2I	905	3.6198	0.0418	Yes	UBC9 — SUMO-conjugating enzyme E2
PPP2CA	930	3.5528	0.0451	Yes	PP2A catalytic subunit α
TRIM8	948	3.5086	0.0486	Yes	TRIM8 E3 ubiquitin ligase
ISG20	992	3.3979	0.0514	Yes	Interferon-stimulated 3′–5′ exonuclease (antiviral)

Supplementary Table 3-E. Reactome "Interferon Signaling" — Core Enrichment (Top 20). Genes listed in this table represent the top 20 contributors to the positive enrichment of the "Interferon Signaling" pathway, one of the most significantly upregulated categories in DGUOK-deficient cells (NES = 2.81, FDR = 6.9×10^{-5}). Upregulated antiviral effectors (*ISG15,*

ISG20, OAS1), signaling mediators (*PLCG1, TRIM8*), and cytoskeletal regulators (*TUBA1A, TUBB2B, TUBB3*) indicate coordinated activation of type I interferon response, cytoskeletal remodeling, and nuclear–cytoplasmic trafficking. These findings correspond with increased ISG mRNA expression validated by RT-qPCR and support a link between disrupted purine metabolism and innate immune activation.

Gene Symbol	Rank in Gene List	Rank Metric Score	Running Enrichment Score	Core Enrichment	Protein Encoded

HMGA1	8	80.4559	0.0008	Yes	High mobility group AT-hook 1 (chromatin architectural protein)
JUN	21	50.0915	0.0015	Yes	c-Jun, AP-1 transcription factor
PSMD2	57	25.0555	0.0015	Yes	26S proteasome non-ATPase subunit 2
CTNNB1	67	21.5850	0.0023	Yes	β-Catenin (Wnt effector/adherens junctions)
FYN	75	19.8539	0.0031	Yes	Src-family tyrosine kinase Fyn
PPP1CB	82	18.7959	0.0040	Yes	Protein phosphatase 1 catalytic subunit β
HGS	96	17.7212	0.0047	Yes	HRS/HGS, ESCRT-0 endosomal adaptor
TAF7	104	17.0915	0.0055	Yes	TFIID subunit TAF7
ACTR3	123	15.0506	0.0060	Yes	ARP3, Arp2/3 actin nucleator
ACTG1	124	15.0269	0.0071	Yes	γ-Actin (cytoskeleton)

ITCH	126	14.6778	0.0081	Yes	ITCH E3 ubiquitin-protein ligase
TUBA1A	157	13.1024	0.0083	Yes	α-Tubulin 1A (microtubules)
AP1S3	167	12.6576	0.0090	Yes	AP-1 complex subunit σ-3 (vesicle trafficking)
BCL2L1	175	12.3372	0.0099	Yes	Bcl-xL, anti-apoptotic regulator
EGFR	192	11.4949	0.0105	Yes	Epidermal growth factor receptor (RTK)
TUBB	194	11.3665	0.0115	Yes	β-Tubulin (class I)
ATP1A1	198	11.3098	0.0125	Yes	Na^+/K^+-ATPase α1 catalytic subunit
CD9	199	11.2676	0.0135	Yes	CD9 tetraspanin (membrane organizer)
ITPR3	222	10.4815	0.0139	Yes	IP3 receptor type 3 (ER Ca^{2+} channel)
SH3KBP1	224	10.4559	0.0149	Yes	CIN85/SH3KBP1 adaptor protein

Supplementary Table 3-F. Reactome “Infectious Disease” — Core Enrichment (Top 20).

This table presents genes from Reactome's "Infectious Disease" module that contribute to positive enrichment in siDGUOK cells. Leading-edge genes such as *JUN*, *CTNNB1*, *ITCH*, *FYN*, and *EGFR* reflect broad activation of host–pathogen defense programs and endosomal trafficking networks. The induction of signaling adaptors (*SH3KBP1, AP1S3*), cytoskeletal components (*ACTR3, ACTG1*), and apoptosis regulators (*BCL2L1*) collectively suggests activation of stress-responsive pathways and cellular remodeling consistent with the interferon-stimulated transcriptional signature.

Gene Symbol	Rank in Gene List	Rank Metric Score	Running Enrichment Score	Core Enrichment	Protein Encoded

LTC4S	28289	-0.4318	-0.3404	Yes	Leukotriene C4 synthase (glutathione-dependent)
PRKAG2	28411	-0.4559	-0.3381	Yes	AMPK γ2 regulatory subunit
ACSBG1	28520	-0.4685	-0.3354	Yes	Very-long-chain acyl-CoA synthetase (bubblegum 1)
CYP4B1	28541	-0.4685	-0.3301	Yes	Cytochrome P450 4B1 (ω-hydroxylase)
ACACB	28694	-0.4949	-0.3287	Yes	Acetyl-CoA carboxylase β (ACC2; FA oxidation control)
ACSL6	28719	-0.4949	-0.3235	Yes	Long-chain acyl-CoA synthetase 6
ACOT6	28736	-0.4949	-0.3180	Yes	Acyl-CoA thioesterase 6
PPT2	28829	-0.5086	-0.3148	Yes	Palmitoyl-protein thioesterase 2 (lysosomal)
HPGD	29048	-0.5528	-0.3154	Yes	15-hydroxyprostaglandin dehydrogenase
SLC27A3	29136	-0.5686	-0.3121	Yes	FATP3, fatty-acid transport protein/acyl-CoA synthetase

SLC25A17	29290	-0.6021	-0.3107	Yes	Peroxisomal transporter PMP34 (CoA/FAD carriers)
ACOT7	29494	-0.6576	-0.3108	Yes	Acyl-CoA thioesterase 7
ELOVL7	29534	-0.6576	-0.3060	Yes	Very-long-chain fatty-acid elongase 7
PCTP	29604	-0.6778	-0.3022	Yes	Phosphatidylcholine transfer protein (StARD2)
HACD2	29798	-0.7212	-0.3020	Yes	3-hydroxyacyl-CoA dehydratase 2 (elongation)
MCAT	30269	-0.8861	-0.3100	Yes	Malonyl-CoA–ACP transacylase (mitochondrial FAS II)
MLYCD	30273	-0.8861	-0.3042	Yes	Malonyl-CoA decarboxylase (regulates FA oxidation)
CYP1A1	30391	-0.9208	-0.3017	Yes	Cytochrome P450 1A1
CYP4F11	30618	-1.0088	-0.3025	Yes	Cytochrome P450 4F11 (ω-hydroxylation of eicosanoids)
FAAH2	30765	-1.0757	-0.3010	Yes	Fatty-acid amide hydrolase 2

Supplementary Table 4-G. Reactome “Fatty Acid Metabolism” — Core Enrichment (Top 20).

The “Fatty Acid Metabolism” pathway was negatively enriched, with leading-edge genes including *ACACB*, *ACSL6*, *ACOT6*, *ELOVL7*, and *FAAH2* showing decreased expression in siDGUOK cells. These genes govern β-oxidation, elongation, and degradation of long-chain fatty acids. Suppression of these transcripts correlates with increased intracellular lipid accumulation and reduced lipid catabolism identified by imaging and biochemical assays. The data reinforce the metabolic phenotype of DGUOK deficiency, linking nucleotide imbalance to impaired lipid utilization.

Gene Symbol	Rank in Gene List	Rank Metric Score	Running Enrichment Score	Core Enrichment	Protein Encoded

MT2A	3	124.6383	0.0013	Yes	Metallothionein-2A (metal/oxidative stress binder)
CXCL8	4	109.3565	0.0026	Yes	Interleukin-8 / CXCL8 chemokine (neutrophil chemoattractant)
IL11	11	76.6990	0.0038	Yes	Interleukin-11 cytokine
JUN	21	50.0915	0.0049	Yes	c-Jun, AP-1 transcription factor
SHC1	32	40.5686	0.0060	Yes	SHC adaptor protein 1 (RTK/STAT signaling adaptor)
GRB10	40	33.0000	0.0072	Yes	GRB10 adaptor (modulates RTK/insulin signaling)
TIMP1	41	31.5086	0.0085	Yes	Tissue inhibitor of metalloproteinases-1
CCND1	44	29.6576	0.0098	Yes	Cyclin D1 (G1/S cell-cycle regulator)
DUSP4	54	25.5229	0.0109	Yes	Dual-specificity phosphatase 4 (MAPK phosphatase)

PSMD2	57	25.0555	0.0122	Yes	26S proteasome regulatory subunit, non-ATPase 2
DUSP6	68	21.2518	0.0133	Yes	MAPK phosphatase 6 (ERK-specific)
FYN	75	19.8539	0.0145	Yes	Src-family tyrosine kinase Fyn
SQSTM1	87	18.6198	0.0155	Yes	Sequestosome-1/p62 (autophagy & NF-κB adaptor)
EGR1	91	18.1427	0.0168	Yes	Early growth response 1 transcription factor
CNN2	94	17.8539	0.0181	Yes	Calponin-2 (actin-binding)
OAS1	148	13.8861	0.0179	Yes	2′-5′-oligoadenylate synthetase 1 (antiviral)
TUBA1A	157	13.1024	0.0190	Yes	Tubulin alpha-1A
JUNB	172	12.4437	0.0200	Yes	JunB, AP-1 transcription factor
BCL2L1	175	12.3372	0.0213	Yes	Bcl-xL (anti-apoptotic mitochondrial protein)

CFL1	227	10.2924	0.0211	Yes	Cofilin-1 (actin depolymerizing factor)

Supplementary Table 3-H. Reactome "Cytokine Signaling in Immune System" — Core Enrichment (Top 20).

This table summarizes the 20 most positively ranked genes driving enrichment of the cytokine signaling pathway. Genes such as *IL11*, *CXCL8*, *JUN*, *EGR1*, *OAS1*, and *TUBA1A* are among the highest contributors, collectively reflecting activation of STAT/MAPK-dependent transcription, chemokine production, and cytoskeletal reorganization. The coordinated upregulation of *GRB10*, *FYN*, and *SHC1* underscores enhanced receptor-tyrosine kinase and cytokine cross-talk, consistent with IFN-β–driven transcriptional reprogramming observed upon DGUOK knockdown. These data demonstrate that purine-driven interferon signaling extends to secondary cytokine cascades influencing cell growth and survival.

Gene Symbol	Rank in Gene List	Rank Metric Score	Running Enrichment Score	Core Enrichment
IDI2	14903	0.00000000	-0.40359333	No

GGPS1	26866	-0.28399664	-0.72023140	No
PLPP6	29259	-0.58502668	-0.75391760	No
LBR	31178	-1.31875873	-0.77358920	Yes
PMVK	31234	-1.35654736	-0.73817830	Yes
ARV1	31829	-1.82390869	-0.71870380	Yes
SREBF1	31866	-1.88605666	-0.68273115	Yes
IDI1	32579	-3.00000000	-0.66674550	Yes
HSD17B7	32804	-3.65757728	-0.63633140	Yes
EBP	32847	-3.82390881	-0.60053610	Yes
SREBF2	32850	-3.82390881	-0.56355820	Yes
TM7SF2	32985	-4.42021656	-0.53048310	Yes
NSDHL	33004	-4.53760195	-0.49397826	Yes
SC5D	33089	-5.04575729	-0.45942482	Yes

CYP51A1	33234	-6.08618593	-0.42664537	Yes
MVK	33250	-6.23657179	-0.39005184	Yes
FDFT1	33515	-10.03151703	-0.36082035	Yes
FDPS	33606	-13.00877380	-0.32644433	Yes
SQLE	33678	-17.25963783	-0.29150650	Yes
DHCR7	33688	-18.30102921	-0.25473556	Yes
MSMO1	33692	-19.00000000	-0.21778722	Yes
DHCR24	33710	-20.22914886	-0.18125282	Yes
ACAT2	33721	-21.30102921	-0.14451145	Yes
MVD	33722	-21.35654640	-0.10747441	Yes
LSS	33751	-25.22184944	-0.07126524	Yes
HMGCR	33807	-36.82390976	-0.03585436	Yes
HMGCS1	33846	-147.55284119	0.00005915	Yes

Supplementary Table 4-I. Reactome "Cholesterol Biosynthesis" Core Enrichment (Top 20)

This table lists the top negatively enriched genes within the Cholesterol Biosynthesis pathway, including SREBF1, PMVK, IDI2, GGPS1, LBR, and ARV1. These enzymes and regulators comprise the mevalonate pathway and sterol-assembly machinery essential for hepatic lipid balance. Their coordinated repression corresponds with decreased sterol and isoprenoid synthesis observed in transcriptomic data, reinforcing that DGUOK deficiency dampens anabolic lipid biosynthesis concurrently with accumulation of neutral lipids and interferon activation.

Gene Symbol	Rank in Gene List	Rank Metric Score	Running Enrichment Score	Core Enrichment	Protein Encoded

OAS1	148	13.88605690	0.0023651960	Yes	2′–5′-Oligoadenylate synthetase 1
TUBA1A	157	13.10237312	0.0088845715	Yes	Tubulin alpha-1A
TUBB2B	255	9.72124672	0.0127630750	Yes	Tubulin beta-2B
TUBB3	269	9.43179798	0.0191340870	Yes	Tubulin beta-3
NUP58	311	8.46852112	0.0246742630	Yes	Nucleoporin 58
UBC	322	8.34678745	0.0311342920	Yes	Polyubiquitin-C
PLCG1	435	6.46852112	0.0345677060	Yes	Phospholipase C gamma-1
TUBB6	444	6.39794016	0.0410870800	Yes	Tubulin beta-6
PPP2R5A	452	6.31875896	0.0476361300	Yes	PP2A regulatory subunit B′ alpha
TUBA1C	469	6.17392540	0.0539181230	Yes	Tubulin alpha-1C
NUP43	519	5.69896984	0.0592209180	Yes	Nucleoporin 43

HSPA2	707	4.30980396	0.060428876<br>0	Yes	Heat shock protein 70-2
TUBA1B	772	4.09151506	0.065286580<br>0	Yes	Tubulin alpha-1B
UBE2I	905	3.61978865	0.068126540<br>0	Yes	UBC9<br>(SUMO-conjugating enzyme E2 I)
PPP2CA	930	3.55284190	0.074171150<br>0	Yes	PP2A catalytic subunit alpha
KPNA4	1026	3.25963736	0.078109000<br>0	Yes	Importin alpha-3
CENPX	1065	3.16749120	0.083738190<br>0	Yes	Centromere protein X
ISG15	1076	3.14266753	0.090198226<br>0	Yes	ISG15 ubiquitin-like modifier
FLNA	1252	2.74472761	0.091762260<br>0	Yes	Filamin-A
UBE2E1	1341	2.58502674	0.095907815<br>0	Yes	Ubiquitin-conjugating enzyme E2 E1

Supplementary Table 4-I. Reactome "Fatty Acid β-Oxidation and Peroxisomal Transport"

This pathway specifically resolves the subset of genes within long-chain β-oxidation and peroxisomal transport processes, separating it from global lipid metabolism. Down-regulated core genes such as SLC27A3, SLC25A17, HACD2, MCAT, and MLYCD indicate suppression of fatty-acid activation, elongation, and CoA transport machinery. Reduced expression of PCTP and HPGD suggests disrupted phospholipid and prostaglandin handling. Collectively, these changes point to impaired peroxisome–mitochondria coordination in energy and lipid homeostasis following DGUOK loss.

Gene Symbol	Rank in Gene List	Rank Metric Score	Running Enrichment Score	Core Enrichment	Protein Encoded
POLR1D	243	10.036	–0.0019	Yes	RNA polymerase I and III subunit D (transcription machinery)
SMARCD1	359	7.538	–0.00005	Yes	SWI/SNF chromatin remodeling complex subunit 1
DR1	601	5.000	–0.0019	Yes	Down-regulator of transcription 1 (NC2β repressor)
UBE2I	905	3.620	–0.0056	Yes	SUMO-conjugating enzyme E2 I (UBC9)
POLR2K	931	3.553	–0.0011	Yes	RNA polymerase II subunit K
SMARCC1	1064	3.170	–0.0021	Yes	SWI/SNF chromatin remodeling complex subunit C1
TAF10	1072	3.153	–0.0022	Yes	TFIID subunit TAF10 (transcription initiation factor)
HAT1	1088	3.115	–0.0024	Yes	Histone acetyltransferase type B catalytic subunit 1
NUP107	1154	2.948	–0.0025	Yes	Nucleoporin 107 (karyopherin complex scaffold)

SUPT5H	1214	2.803	–0.0026	Yes	Transcription elongation factor Spt5
RUVBL2	1232	2.764	–0.0027	Yes	AAA+ ATPase component of chromatin remodeling complexes
H2AFZ	1242	2.742	–0.0028	Yes	Histone variant H2A.Z (promotes nucleosome mobility)
SUPT16H	1285	2.656	–0.0029	Yes	FACT complex subunit Spt16 (histone chaperone)
TAF9	1310	2.608	–0.0030	Yes	TFIID subunit TAF9 (transcription initiation factor)
RUVBL1	1321	2.586	–0.0031	Yes	AAA+ ATPase RuvB-like 1 (chromatin remodeler)
CHD1	1333	2.562	–0.0031	Yes	Chromodomain-helicase-DNA-binding protein 1 (remodeler)
TAF5	1339	2.550	–0.0032	Yes	TFIID subunit TAF5 (core scaffold component)
ING3	1343	2.542	–0.0033	Yes	Inhibitor of growth family member 3 (NuA4 complex subunit)
RBBP7	1349	2.530	–0.0033	Yes	Histone-binding protein RbAp46/48 (chromatin assembly factor)

EP400	1352	2.524	–0.0034	Yes	E1A-binding protein p400 (Tip60 HAT complex component)

Supplementary Table 4-J. Reactome "Epigenetic Regulation of Gene Expression"

This table lists the top 20 leading-edge genes contributing to enrichment of the Reactome *"Epigenetic Regulation of Gene Expression"* pathway identified by GSEA preranked analysis comparing **siDGUOK** and **siCON HEPG2** cells. Core genes such as *SMARCD1*, *RUVBL1*, *RUVBL2*, *EP400*, and *CHD1* encode components of chromatin-remodeling and transcriptional coactivator complexes including SWI/SNF, NuA4, and TFIID. The listed genes exhibit positive rank-metric scores, reflecting relative upregulation in the siDGUOK condition. Enrichment within this pathway indicates increased representation of factors involved in histone modification, nucleosome remodeling, and transcriptional regulation under the analyzed condition.

Gene Symbol	Rank in Gene List	Rank Metric Score	Running Enrichment Score	Core Enrichment
POLR1D	243	10.0362	−0.0019	Yes
SMARCD1	359	7.5376	−0.00005	Yes
DR1	601	5.0000	−0.0019	Yes
UBE2I	905	3.6198	−0.0056	Yes
POLR2K	931	3.5528	−0.0011	Yes
SMARCA4	1078	3.1367	−0.0001	Yes
RBM7	1103	3.0605	0.0044	Yes
DEK	1129	3.0000	0.0090	Yes
ATF7IP	1143	2.9586	0.0139	Yes
SPOCD1	1347	2.5686	0.0132	Yes
SUMO2	1364	2.5376	0.0180	Yes
ZNF765	1607	2.1938	0.0161	Yes
ZNF669	1631	2.1739	0.0207	Yes

ACTB	1749	2.0605	0.0225	Yes
POLR2H	1826	2.0000	0.0255	Yes
ZNF264	2269	1.6021	0.0177	Yes
SS18L1	2424	1.5229	0.0184	Yes
SAP30	2453	1.5086	0.0229	Yes
SETD1B	2542	1.4559	0.0255	Yes
MTA2	2606	1.4202	0.0290	Yes

Supplementary Table 4-J. Reactome "Epigenetic Regulation of Gene Expression"

This table lists the top 20 leading-edge genes contributing to enrichment of the Reactome "Epigenetic Regulation of Gene Expression" pathway identified by GSEA preranked analysis comparing siDGUOK and siCON HEPG2 cells. Core enriched genes include POLR1D, SMARCD1, SMARCA4, UBE2I, and SETD1B, which encode components of chromatin-remodeling, histone-modifying, and transcriptional co-regulatory complexes. The listed genes exhibit positive rank-metric scores, indicating relative upregulation in the siDGUOK condition. Enrichment within this pathway denotes increased representation of genes associated with histone modification, nucleosome remodeling, and transcriptional regulation in the analyzed comparison.

Gene Symbol	Rank in Gene List	Rank Metric Score	Running Enrichment Score	Core Enrichment
MRI1	1137	2.9586	0.1331	Yes
MTAP	1478	2.3566	0.2897	Yes
ENOPH1	2041	1.7696	0.4398	Yes
GOT1	2942	1.2366	0.5798	Yes
APIP	8122	0.3010	0.5935	Yes
ADI1	33585	−12.3565	0.0078	No

Supplementary Table 4-K. Reactome "Methionine Salvage Pathway"

This table lists the leading-edge genes contributing to enrichment of the Reactome *"Methionine Salvage Pathway"* identified by GSEA preranked analysis comparing siDGUOK and siCON HEPG2 cells. Core enriched genes such as *MRI1*, *MTAP*, *ENOPH1*, *GOT1*, and *APIP* encode enzymes involved in the methionine salvage and transsulfuration cycles. The listed genes exhibit positive rank-metric scores, indicating relative upregulation in the siDGUOK condition. Enrichment within this pathway reflects increased representation of genes involved in methionine recycling, polyamine metabolism, and related sulfur-amino-acid processes under the analyzed comparison.

Gene Symbol	Rank in Gene List	Rank Metric Score	Running Enrichment Score	Core Enrichment
PNP	84	18.7212	0.0083	No
GLRX	133	14.4559	0.0176	No
GUK1	289	8.9208	0.0238	No
CDA	400	6.7959	0.0312	No
GART	748	4.1739	0.0317	No
AK6	874	3.6990	0.0388	No
NME2	897	3.6576	0.0489	No
DCTPP1	1043	3.2366	0.0553	No
TXNRD1	1107	3.0555	0.0642	No
NME3	1377	2.5086	0.0670	No
TXN	1496	2.3279	0.0743	No
RRM2	1569	2.2366	0.0829	No
DCK	1650	2.1487	0.0913	No

NME1	1688	2.1192	0.1009	No
GMPR2	1703	2.0969	0.1113	No
ENTPD6	1893	1.9208	0.1164	No
NUDT15	1937	1.8539	0.1259	No
CTPS1	2048	1.7696	0.1334	No
AK2	2103	1.7213	0.1425	No
AMPD3	2169	1.6778	0.1514	No

Supplementary Table 4-L. Reactome "Metabolism of Nucleotides"

This table lists the top 20 ranked genes contributing to the Reactome *"Metabolism of Nucleotides"* pathway identified by GSEA preranked analysis comparing siDGUOK and siCON HEPG2 cells. Genes such as *PNP*, *GUK1*, *CDA*, *NME1*, and *CTPS1* encode enzymes involved in nucleotide interconversion, deoxyribonucleotide synthesis, and purine–pyrimidine metabolism. The rank-metric scores are positive, indicating relative upregulation in the siDGUOK condition, while none of the listed genes were designated as core enriched within the leading-edge subset. This enrichment profile represents the relative contribution of nucleotide metabolic genes to the ranked gene list within the analyzed comparison.

Gene Symbol	Rank in Gene List	Rank Metric Score	Running Enrichment Score	Core Enrichment
PNP	84	18.7212	0.0269	No
ENTPD6	1893	1.9208	0.0029	No
NUDT15	1937	1.8539	0.0310	No
ITPA	3021	1.2076	0.0284	No
ADPRM	3326	1.0809	0.0488	No
NT5C	4301	0.7959	0.0494	No
UPP1	4343	0.7959	0.0776	No
ENTPD4	4563	0.7447	0.1006	No
NUDT18	5746	0.5376	0.0950	No
DPYS	9967	0.1805	−0.0004	No
UPP2	10898	0.1308	0.0015	No
NT5C3A	11717	0.1079	0.0068	No
ENTPD1	11954	0.0915	0.0292	No

TYMP	12159	0.0757	0.0526	No
ENTPD3	12696	0.0506	0.0661	No
XDH	15031	0.0000	0.0265	No
NT5C1B	15754	0.0000	0.0346	No
NUDT5	25301	−0.1308	−0.2183	No
NT5M	25819	−0.1739	−0.2042	No
AGXT2	27221	−0.3188	−0.2162	No

Supplementary Table 4-M. Reactome "Nucleotide Catabolism"

This table lists the top 20 ranked genes contributing to the Reactome *"Nucleotide Catabolism"* pathway identified by GSEA preranked analysis comparing **siDGUOK** and **siCON HEPG2** cells. Genes such as *PNP*, *ITPA*, *ADPRM*, *ENTPD* family members, and *NT5C* encode enzymes involved in nucleotide dephosphorylation, base salvage, and degradation. All listed genes exhibit a range of positive to negative rank-metric scores, indicating variable relative expression across the ranked gene list. None of the genes in this set were designated as core enriched, and the table reflects the distribution of catabolic enzymes contributing to overall pathway representation.

siRNA	Vendor	Catalog Identifier
siDGk-Pooled	Dharmacon/Horizon Discovery	M-006719-03-0010
siDGk- Set of 4	Dharmacon/Horizon Discovery	MQ-006719-03-0010
siIFNAR1	Dharmacon/Horizon Discovery	M-020209-00-0010
siTMEM173	Dharmacon/Horizon Discovery	M-024333-00-0010
siRELA	Dharmacon/Horizon Discovery	M-003533-02-0010
siDDX58	Dharmacon/Horizon Discovery	M-012511-01-0010
siIFNGR1	Dharmacon/Horizon Discovery	M-011057-00-0010

Supplementary Table 5. siRNA Reagents

Commercially sourced siRNA reagents used for DGUOK knockdown, deconvolution analysis, and IFN/innate immune pathway silencing are listed by vendor and catalog identifier. The pooled siDGUOK reagent was used for primary knockdown experiments, while individual siRNAs from the siDGUOK set of 4 were tested separately to assess whether independent DGUOK-targeting siRNAs reproduced the molecular and lipid phenotypes observed with pooled siDGUOK.

siRNA	Target Sequences
siDGk1	GCACGAUGGUCCUACACAU
siDGk2	UUGGAAACUUGCUGGAUAU
siDGk3	GGUGUUGGAUGUCAAUGAU
siDGk4	ACAAGAAGACCUCAUGAGA

Supplementary Table 6. Individual siDGUOK target sequences from the siDGUOK set of 4.

Target sequences are shown for the four individual siRNAs included in the commercial siDGUOK set of 4. For deconvolution analysis, siDGk1 and siDGk2 were tested individually to assess whether independent DGUOK-targeting siRNAs reproduced the molecular and lipid phenotypes observed with the pooled siDGUOK reagent.
